# Wide-field optical coherence tomography for microstructural analysis of key tissue types: a proof-of-concept evaluation

**DOI:** 10.3389/pore.2023.1611167

**Published:** 2023-07-14

**Authors:** Beryl Rabindran, Adriana D. Corben

**Affiliations:** ^1^ Perimeter Medical Imaging AI, Toronto, ON, Canada; ^2^ Icahn School of Medicine at Mount Sinai, Mount Sinai Hospital, New York, NY, United States

**Keywords:** histopathology, breast-conserving surgery, histology, optical coherence tomography, intraoperative margin assessment

## Abstract

**Introduction:** The presence of positive margins following tumor resection is a frequent cause of re-excision surgery. Nondestructive, real-time intraoperative histopathological imaging methods may improve margin status assessment at the time of surgery; optical coherence tomography (OCT) has been identified as a potential solution but has not been tested with the most common tissue types in surgical oncology using a single, standardized platform.

**Methods:** This was a proof-of-concept evaluation of a novel device that employs wide-field OCT (WF-OCT; OTIS 2.0 System) to image tissue specimens. Various cadaveric tissues were obtained from a single autopsy and were imaged with WF-OCT then processed for permanent histology. The quality and resolution of the WF-OCT images were evaluated and compared to histology and with images in previous literature.

**Results:** A total of 30 specimens were collected and tissue-specific microarchitecture consistent with previous literature were identified on both WF-OCT images and histology slides for all specimens, and corresponding sections were correlated. Application of vacuum pressure during scanning did not affect specimen integrity. On average, specimens were scanned at a speed of 10.3 s/cm^2^ with approximately three features observed per tissue type.

**Conclusion:** The WF-OCT images captured in this study displayed the key features of the most common human tissue types encountered in surgical oncology with utility comparable to histology, confirming the utility of an FDA-cleared imaging platform. With further study, WF-OCT may have the potential to bridge the gap between the immediate information needs of the operating room and the longer timeline inherent to histology workflow.

## Introduction

Surgical excision remains the first-line treatment for localized, early-stage solid tumors arising from cancers of the breast, colon and rectum, kidney, lung, thyroid, and those of other tissues and organs [1, 2]. Its effectiveness is dependent upon the complete removal of diseased tissue, ideally encapsulated within a safe margin of healthy, normal tissue. In breast-conserving surgery (BCS), for example, histopathologically “clear” or “negative” tumor margins have been associated with a twofold decrease in tumor recurrence [3, 4], which in turn has a significant favorable impact on overall morbidity, mortality, and the economics of managing breast cancer [3–5].

When, on pathological examination, cancerous cells are found at or near the margin of the tumor, additional tissue must be removed from the resection bed to reduce the risk of disease recurrence [6]. For example, in BCS, re-excision for positive or close margins is indicated when disease is detected within 2 mm from the tumor surface for *in situ* disease, and less than 1 mm for invasive disease [4, 5, 7]. Unfortunately, the typical timeframe for standard-of-care histological analysis of excised tissue is on the order of days to weeks; therefore, a positive margin generally necessitates a return to the operating room for a second surgery. Re-excision has been estimated to occur in greater than 20% of cases on average and has been independently associated with poorer clinical outcomes, higher costs of care, and reduced patient satisfaction with treatment [8–12].

Methods that can provide a rapid, thorough, and non-destructive intraoperative assessment of excisional tissue margins have the potential to improve the overall success of primary tumor resection, minimize the incidence of re-excision surgeries and local tumor recurrence, and improve costs of care [6]. Their development is, accordingly, of paramount importance for patients, surgeons, healthcare systems, and payors [13].

Optical coherence tomography (OCT) is one of the oldest and most extensively studied tomographic modalities for imaging of human tissues on a microscopic scale [14]. First described for imaging of the retina and coronary arteries in the 1990s [15], OCT uses near infra-red interferometry to produce cross-sectional images of biological tissues. The underlying principle is analogous to ultrasound imaging; in the case of OCT, light (1,250–1,350 nm) responds to differences in refractive index and optical scattering and absorption properties associated with the structures found in biological tissues [14, 16, 17]. The echo and time delay of the reflected, backscattered light are exploited to generate images with an axial resolution of 6–15 μm at a penetration depth up to 2 mm. Thus, OCT facilitates rapid, non-destructive visualization of clinically relevant tissue microarchitecture at or near the surface of a tissue sample without sectioning, labeling, or other preparation. OCT has a long history of use in ophthalmology [14, 18], and has been adapted to intravascular assessment of atherosclerotic disease [14, 16, 19].

In 2016, a wide-field optical coherence tomography (WF-OCT) imaging platform (OTIS 1.0 System, Perimeter Medical Imaging AI, Inc. Toronto, Canada) was cleared to market by the US FDA for general imaging of tissue microstructure. With WF-OCT a user can view real-time, high-resolution, cross-sectional tissue images up to 2 mm in depth from the tissue surface. Image resolution is approximately 30 µm and imaging requires no use of chemicals or radiolabeling and does not affect the sample quality for subsequent histology [20–23]. The system has previously demonstrated the ability to evaluate tissue specimens in oropharyngeal cancer resections prior to routine pathology, including margin assessment without specimen damage or disruption of workflow, in addition to utility in imaging the margin of resected breast tissue at the time of surgery [20–23, 23, 24]. Other studies of the system with single and multiple readers have demonstrated a high degree of concordance between breast tissue margin assessment based on WF-OCT and the corresponding histology slides, with greater than 85% sensitivity and specificity for identifying tissue changes suspicious for malignancy. There remains, however, a greater need to investigate the comparability of WF-OCT images to histology in more tissue types, in both normal and pathological states, on a single, standardized platform. Accordingly, we conducted a proof-of-concept study to assess whether WF-OCT images from the system had sufficient quality and resolution to identify tissue microstructures unique to the most common tissue types encountered in surgical oncology, as assessed by qualified clinicians.

## Materials and methods

This was a proof-of-concept study using a commercial WF-OCT system on multiple tissue types obtained from a single autopsy. The study objective was to assess whether images generated by the system were sufficiently detailed to allow qualified clinician reviewers to resolve near-surface tissue microstructures on the order of 50 μm, identify key strata therein (e.g., epithelial, adipose, and stromal tissue), and correlate them to histology slides of the same sample. The study was also intended to investigate whether or not specimen integrity and viability for standard pathology processing were compromised during system use, and that the user interface adequately enabled the reviewers to interact with acquired scans.

### Ethics

The deidentified human tissue used for this study was procured via autopsy by the Biorepository of the Icahn School of Medicine at Mount Sinai Hospital (New York, NY, United States), from a cadaver donated for research purposes. Ethical approval was obtained by the Biorepository at the Icahn School of Medicine for the use of the human cadaver in this study, including informed written consent from the legally authorized representative. The protocol was granted an exemption from ethics approval by the Institutional Review Board of Mount Sinai Hospital. The study sponsor did not have access to protected health information.

### WF-OCT system

A newer version of the original system was used in this study (OTIS 2.1 System); the device received FDA 510(k) clearance in 2021 for use as an imaging tool in the evaluation of excised human tissue microstructure by providing two-dimensional, cross-sectional, real-time depth visualization, with image review manipulation software for identifying and annotating regions of interest. The device can obtain OCT images with sufficient image quality to identify tissue microstructure in thyroid, colon, ovary, uterus, cervix, skin and breast tissue; however, the system has a general indication in the US and is not specifically indicated for use in any tissue types. The system comprises a console for specimen scanning and a disposable component for specimen handling. The console is a mobile cart that provides automated OCT scanning of individual margins of the excised specimen. To scan, the user positions the specimen against the flat, OCT-transparent imaging window of the consumable set. A user-adjustable level of vacuum is applied to secure the tissue in place. The vacuum pressure also reduces the gap between the glass surface and the tissue, thereby ensuring good image quality. The scanner employs an automated image probe positioning mechanism to enable rapid capture of multiple, small, conventional OCT images from excised tissue surfaces. The software tiles these images into stacks for review by the user, providing microscopic visualization of cross-sectional images at a depth of up to 2 mm below the surface of a sample, with a maximum size of 9 cm × 9 cm at approximately 30 µm resolution.

The console has a user interface system with a touch screen that allows for data input and clinician review of collected images. The OCT images are paired with photographic surface images via review software that allows clinicians to identify, manipulate, and annotate regions of interest.

### Study procedures

#### Specimen preparation, imaging, and histology

The study was conducted at Mount Sinai Hospital on excised, cadaveric human tissue retained for research purposes and procured within 24 h following death.

The specimen preparation workflow is summarized in Supplementary Figure S1. Tissue samples were resected and grossed using standard methodology at the autopsy laboratory. A total of three specimens were excised for each tissue type. For WF-OCT image acquisition, each sample was dotted with specimen ink to mark orientation, placed in a disposable tray, and positioned on the specimen window. Each specimen was then photographed for documentation purposes and secured in place using either low, high, or no vacuum pressure to compare the effect of vacuum on specimen integrity (one specimen per tissue type at each vacuum setting). After scanning, each specimen was again photographed and palpated by the pathologist to assess tissue integrity after vacuum application.

Following photography and OCT image capture, each of the three specimens was fully inked, grossed, placed in a specimen cassette, and fixed in neutral buffered formalin. Specimens were then transported to the Biorepository CoRE lab at Mount Sinai Medical Center, where they were processed into slides, stained with hematoxylin and eosin (H&E), and then digitized for further analysis.

#### Specimen review and correlations

A pathologist from Mount Sinai Hospital (ADC) reviewed and annotated the digitized histology slides using CaseViewer (version 2.2, 3DHISTECH Ltd., Budapest, Hungary), noting the microstructures and other features pertinent to each specific tissue type.

Separately, WF-OCT images were reviewed and annotated by a lead clinical scientist (BR) from the study sponsor, using the OTIS software (version 2.0.10, loaded on a Windows-based workstation), as well as a clinical leader from the sponsor to validate the annotations. To assist review, the clinical scientist used reference images from a number of prior publications showing correlations between swept-source or other OCT approaches and histology for some of the tissue types used in this study [25–32].

Once the separate reviews were complete, the histology slides were correlated with the OCT images that displayed corresponding layers and microstructures and the B-scan number (optical “slice”) of each OCT image was noted. This correlation work enabled the creation of an atlas of the appearance of tissue-specific layers and microstructures in the OCT images.

### Data analysis

This was a proof-of-concept evaluation. The time taken to scan each tissue specimen as well as the sizes of the specimens were noted (Table 1). The features observed in each tissue type were tabulated and displayed descriptively. The features observed in reference images [25–32] were also tabulated, providing a side-by-side comparison of previous studies with this device (Table 2).

**TABLE 1:** Specimen sizes and scan times.

Tissue type	#	Vacuum level	Specimen sizes (cm) L, B, W			Scan area selected (cm) L, B		Total scan area (cm^2^)	Scan time (sec)	Scan time/area (sec/cm^2^)
			L	B	W	L	B			
Breast	1	Control	5	5	0.5	N/A	N/A	N/A	N/A	N/A
		Low	5	7	0.5	2	3	6	58	9.67
		High	4.5	6	0.5	2	2	4	39	9.75
Heart	2	Control	6	2	0.5	N/A	N/A	N/A	N/A	N/A
		Low	5	3	0.5	2	2	4	39	9.75
		High	6	3	1	2	1	2	20	10.00
Kidney	3	Control	3.5	2.5	1	N/A	N/A	N/A	N/A	N/A
		Low	3	1.5	1	2	1	2	20	10.00
		High	3	1.5	0.5	2	1	2	20	10.00
Spleen	4	Control	8	6.5	1	N/A	N/A	N/A	N/A	N/A
		Low	8	5	1	3	3	9	86	9.56
		High	8	5	1	3	3	9	86	9.56
Thyroid	5	Control	2	1.5	0.5	N/A	N/A	N/A	N/A	N/A
		Low	4	1.8	0.5	1	3	3	30	10.00
		High	2	1	0.5	1	2	2	20	10.00
Adrenal	6	Control	2	1.5	0.5	N/A	N/A	N/A	N/A	N/A
		Low	2.5	2	0.5	2	2	4	39	9.75
		High	3	2.5	0.5	1	2	2	20	10.00
Pancreas	7	Control	3.5	2.5	1	N/A	N/A	N/A	N/A	N/A
		Low	2.5	3.5	1	1	2	2	39	19.50
		High	6.5	2	2	2	2	4	39	9.75
Liver	8	Control	10	4.5	1.5	N/A	N/A	N/A	N/A	N/A
		Low	10	7	1	3	3	9	86	9.56
		High	11	6.5	1	3	3	9	86	9.56
Lung	9	Control	2	3	0.5	N/A	N/A	N/A	N/A	N/A
		Low	3.5	2	0.5	2	2	4	39	9.75
		High	3.5	2.5	0.5	2	2	4	39	9.75
Colon	10	Control	6	2	0.3	N/A	N/A	N/A	N/A	N/A
		Low	7.5	3.5	0.3	2	2	4	39	9.75
		High	4.5	2	0.5	3	2	6	58	9.67
Average			**5.03**	**3.31**	**0.74**	**2.05**	**2.15**	**4.55**	**45.10**	**10.27**

The table above shows the sizes of the different tissue specimens scanned. The scan areas and scan times for the specimens scanned on low and high vacuum pressure are shown as well. The control specimen was not scanned and used as a control to compare specimen integrity with the specimens scanned on low and high vacuum.

Tissue type: Type of tissue scanned using WF-OCT; #: Number; Vacuum level: Vacuum setting on WF-OCT device used to secure the specimen in place; Specimen sizes (cm) L, B, W: Size of specimen scanned using WF-OCT. L-Length, B-Breadth, W- Width; Scan area selected (cm) L, B: Dimensions of scan area selected on the WF-OCT device for scanning. L- Length, B-Breadth; Total scan area (cm^2^): L*B from column 5 (scan area selected); corresponds to the total area scanned on the specimen; Scan time (sec): Time taken to scan this area on the specimen; Scan time/area (sec/cm^2^): Time taken to scan per cm^2^ of the specimen.

**TABLE 2:** Summary of features resolvable in reference publications and WF-OCT.

#	Tissue	References	Layers/Features resolved in literature	Layers/Features resolved in WF-OCT	Number of features resolved in literature	Number of features resolved in WF-OCT
1	Breast	[27]	Adipose tissue, Fibrous tissue (stroma), Duct	Adipose tissue, Fibrous tissue (stroma), Duct	3	3
2	Heart	[26]	Adipose Tissue, Collagen, Myocardium	Adipose tissue, Muscle	3	2
3	Kidney	[31]	Glomerulus, Tubules	Glomerulus, Vessel	2	2
4	Thyroid	[25]	Follicles, Stroma, Capsule	Follicles, Stroma, Capsule, Cyst, Vessel	3	5
5	Pancreas	[29]	Cysts	Vessel, Adipose tissue, Parenchyma	1	3
6	Liver	[32]	Vessel, Connective tissue layers, Serosa, Epithelium	Vessel, Fibrosis, Duct	4	3
7	Lung	[28]	Necrosis, Fibrosis	Vessel, Alveoli	2	2
8	Colon	[30]	Upper-Mucosa, Muscularis Mucosae, Sub-Mucosa, Muscularis propria, Crypts	Upper-Mucosa, Sub-Mucosa, Muscularis propria	5	3
	Average				2.875	2.875

The table above provides a summary of the features observed in each of the tissue types against those in reference publications. It also shows a comparison of the number of features observed in literature for each tissue type and the number of features observed using WF-OCT. Two tissue types were not included in the table because no reference publications showing the OCT images for these tissue types were available—Spleen and Adrenal Glands.

## Results

### Tissue preparation, OCT imaging, and tissue integrity

All tissues for this study were excised from a deceased, 68-year-old female donor secondary to hemorrhagic and septic shock from a bacterial infection. Her past medical history was significant for renal failure, liver cirrhosis, and emphysema. Tissue specimens were excised and imaged within 24 h of death.

Ten tissue types were scanned from this patient, with three specimens taken per tissue type. These three specimens were scanned at no vacuum, low vacuum and high vacuum pressure respectively, for each tissue type. Table 1 shows the sizes of all 30 specimens, along with the scan areas of the specimens scanned at low and high vacuum. The average size of the specimen scanned was 5.0 cm × 3.3 cm × 0.74 cm. For an average scan area of 2.0 × 2.2 cm^2^, the average scan time was 45.1 s, at the rate of 10.3 s/cm^2^.

All 30 of the specimens included in the primary evaluation were successfully scanned and processed. The pathologist confirmed that specimen integrity and viability for routine pathology processing was not compromised when specimens were scanned under vacuum at any setting, showing that using vacuum is safe for specimen scanning using this WF-OCT device for the ten tissue types scanned in this study. Images of the gross, unprocessed specimens before and after OCT scanning are displayed in the Supplementary Material.

For all 10 tissue types, we looked at previous literature to determine what other features had been observed in similar studies using OCT [25–32]. We tabulated the features seen previously using OCT against the features we saw using WF-OCT (Table 2). We found references for eight tissue types; spleen and adrenal glands did not have any prior publications showing OCT images. For the eight tissue types that we compared, on average 2.875 features/tissue type were observed in literature. This was the same as the average number of features observed in WF-OCT, with no significant difference in the number of features observed in previous studies vs. in this study (*p* > 0.05).

### Comparative histology

The pathologist confirmed that the appearance of both healthy and diseased tissue in the histology slides was as expected in clinical practice, given the tissue donor’s health status at time of death. Further, the observed tissue architecture was comparable between the WF-OCT images and the corresponding histology, both for healthy and diseased tissue types.

**FIGURE 1:**
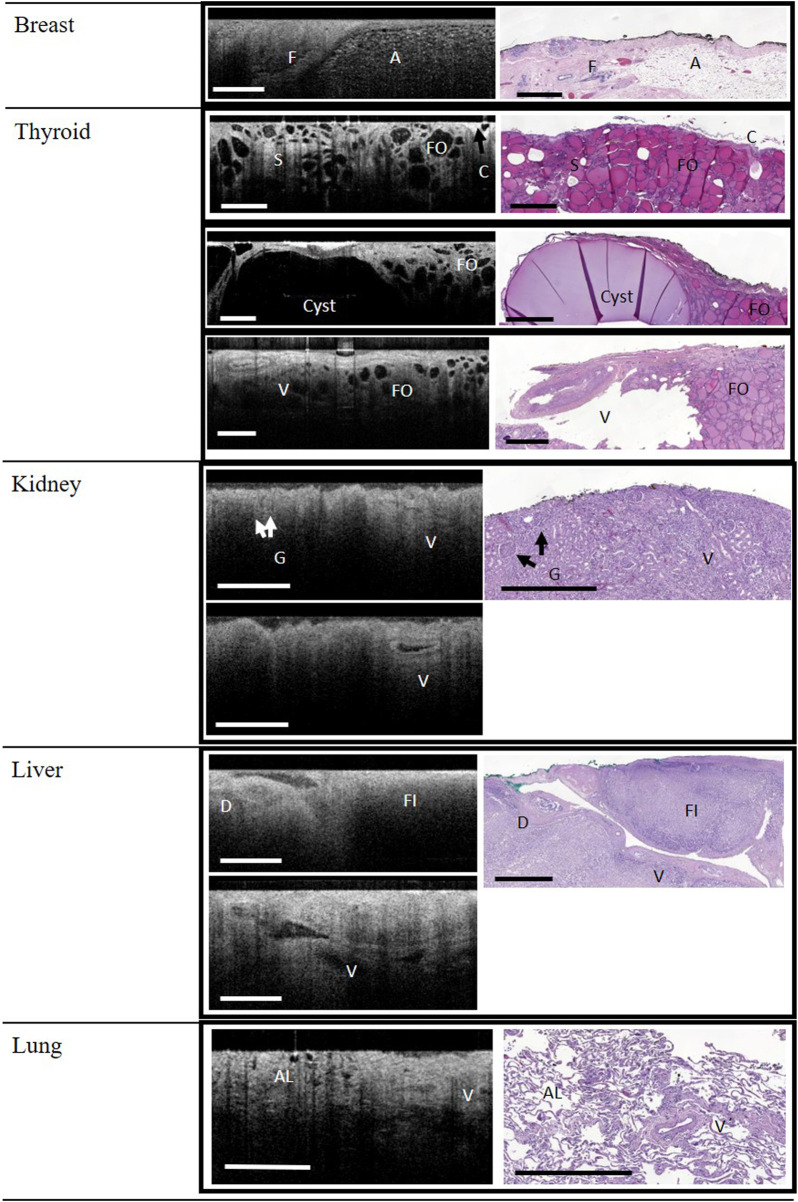
WF-OCT images and correlated histology from Breast, Thyroid, Kidney, Liver and Lung. Reference images for kidney and liver (lower panels) demonstrate that vessels (V) could be followed across multiple WF-OCT image slices. Abbreviations: A, adipose tissue; C, Capsule; F, fibrous tissue; FO, follicle; S, Stroma; AL, alveoli; D, Duct; FI, fibrosis; G, glomerulus; V, vessel; WF-OCT, wide-field optical coherence tomography. Scale bar: 1 mm.

**FIGURE 2:**
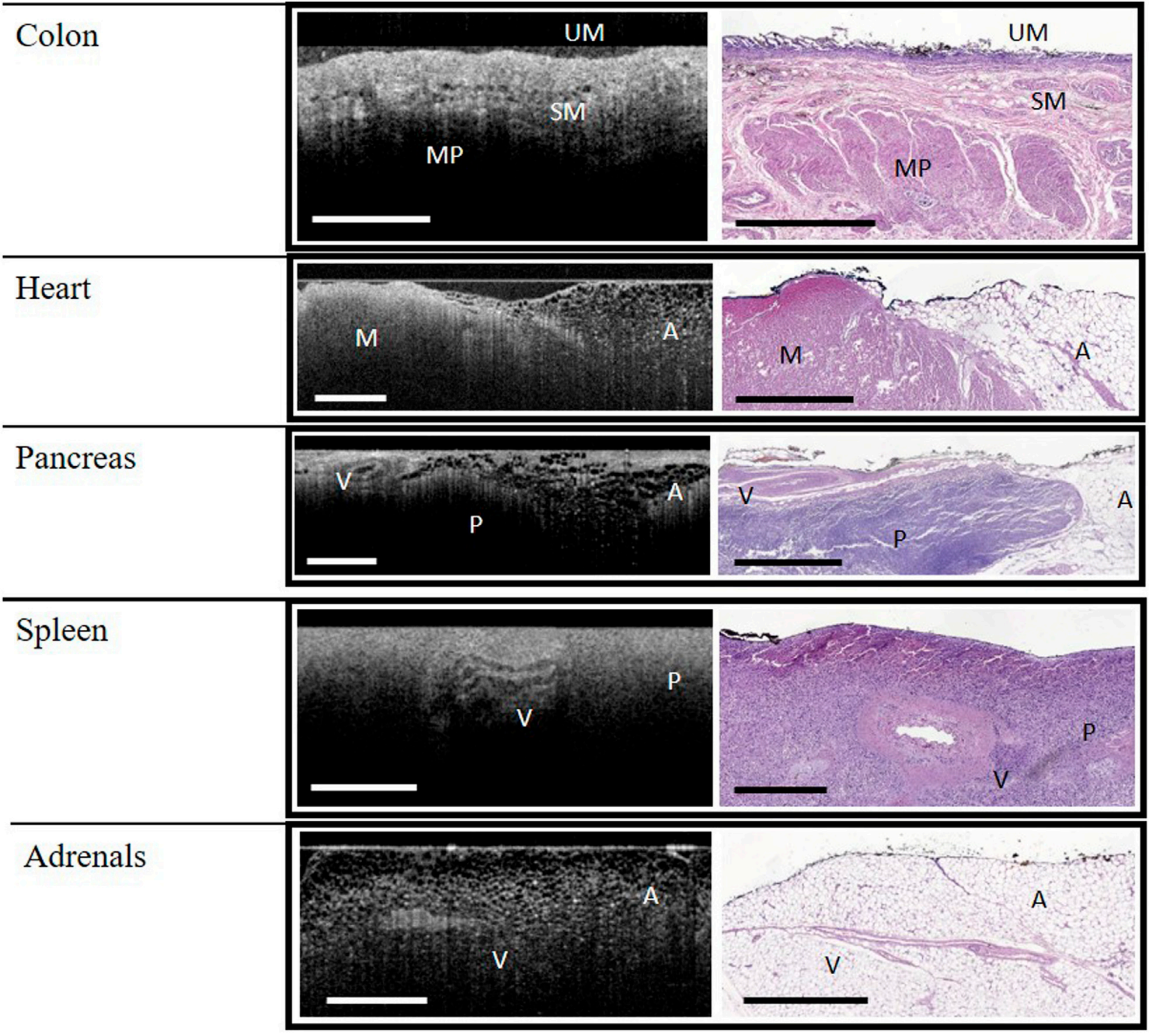
WF-OCT images and correlated histology from Colon, Heart, Pancreas, Spleen and Adrenal Glands. Abbreviations: A, adipose tissue; M, muscle; MP, muscularis propria; P, parenchyma; SM, submucosa; UM, upper mucosa; V, vessel; WF-OCT, wide-field optical coherence tomography. Scale bar: 1 mm.

Representative image correlations between the WF-OCT images and histology slides from each tissue type are displayed in Figures 1, 2. Adipose tissue, fibrous tissue (stroma), and ducts were observed in the breast tissue in both WF-OCT images and histology slides (Figure 1). The thyroid specimens displayed follicles, stroma, capsule, a cyst, and a vessel (Figure 1).

Glomeruli and vessels could be observed in the kidney specimens, both by histology and WF-OCT (Figure 1). Tubules were not observed with WF-OCT, and histology showed dying/necrotic tubules with lack of discernible nuclei, consistent with renal failure. The liver was fibrotic due to cirrhotic disease, and although the fibrotic tissue structure could be observed in both histology and WF-OCT, neither method was able to detect connective tissue nor serosa layers. However, vessels and ducts were observed in both histology and WF-OCT images (Figure 1). Both WF-OCT and histology images displayed normal lung features such as alveoli and vessels, with evidence of localized inflammation and damaged alveoli characteristic of emphysema (Figure 1).

In the colon specimens, the upper mucosa, submucosa, and muscularis propria layers were identified in both histology and WF-OCT images (Figure 2). Crypts were not observed by either method, and a lower-than-expected density of glands on the tissue surface was attributed to autolysis. While both adipose and muscle tissues were observed in the heart specimens, collagen and myocardium layers were not observed by either method (Figure 2). Features observed in the pancreas specimens, by both histology and WF-OCT imaging, were adipose tissue, parenchyma, and vessels (Figure 2).

The features observed in both histology and WF-OCT images of the spleen were vessels and parenchyma. Vessels and adipose tissue were observed in the adrenal tissue (Figure 2).

### General usability and image quality

A notable feature of the WF-OCT analysis on this system was the ability to “scroll” between multiple image slices rapidly, in essence allowing the evaluator to visualize microstructures through a 3-dimensional volume of tissue. Indeed, as shown in Figures 1, 2, the pathway of vessels in thyroid, kidney, and liver specimens could be followed through the specimen. Video examples of this feature are shown in Supplementary Videos S1–S3.

The evaluators noted an anecdotal observation that the quality of the WF-OCT images acquired for this study were at least comparable to and in most cases of higher quality than those generated by swept-source devices as found in the literature [25–32], and overall image quality contributed to the ease of interpretation of the images.

## Discussion

In this proof-of-concept study of a standardized platform for WF-OCT imaging, trained clinicians were consistently able to identify normal and pathologic tissue-specific layers, features, and microstructures in 10 different tissue types, collected via autopsy of a donor with a known clinical course. In some cases—especially in the kidneys, liver, and lungs—the tissue microarchitecture was observably abnormal, secondary to ongoing disease processes at the donor’s time of death; however, it is notable that by histology, the observed changes were consistent with the clinical course and that they could be correlated to corresponding features observed in the WF-OCT images. In other words, the WF-OCT images and histology slides demonstrated both normal and pathological tissue features consistent with the donor’s clinical history. Thus, the objectives of the study were met.

Although none of the tissue types analyzed in the present study were malignant, this study has important implications for the use of WF-OCT as an adjunctive imaging technique for real-time review of tissue microarchitecture. At this time, there are a number of adjunctive techniques and technologies for assessing intraoperative margin status, but each has limitations that affect overall utility for clinical management [6, 33]. For example, while intraoperative frozen section analysis has demonstrated high sensitivity and specificity, the technique is costly, technically difficult, and may compromise the sample’s integrity for histology [6, 33]. Imprint cytology is moderately sensitive and specific but requires specialized expertise in sample preparation and interpretation and is not able to distinguish *in situ* vs. invasive disease, or measure margin depth [33]. Specimen radiography and intraoperative ultrasound have also demonstrated high intraoperative utility, however neither is able to reliably detect noncalcified nor nonpalpable lesions, respectively, and microscopic analysis at the margin is impossible with these methods [6, 33]. Finally, although a radiofrequency spectroscopy device (Margin Probe, Dune Medical, Alpharetta, Georgia) was previously cleared by the FDA to differentiate benign from malignant breast tissue at the margin, the device’s pivotal trial to market clearance demonstrated that the low overall specificity (46.4%; 95% CI: 42.9–49.9) of the technology led to a tripling of the false-positive rate (53.6% vs. 16.6% for the device and control arms, respectively) and an unacceptably high number of unnecessary cavity shaves to remove additional tissue at the margins [34, 35].

Already in use for ophthalmic and intravascular clinical and translational applications [36–39], OCT capitalizes on the light-scattering properties of biologic tissues to generate cross-sectional images of microscopic features and structures, in both nontransparent and transparent tissues [17, 39]. There are several important rationales for using this technique to assess tumor surface features during surgery. First, OCT specimen preparation is nondestructive and label-free, and does not expose patients or clinicians to ionizing radiation. Second, images are generated in real time, at a clinically relevant depth and resolution [6, 17]. Lastly, because OCT is non-destructive, it does not affect downstream histology, and as shown in this report and others, the images generated by WF-OCT can be matched, correlated to, and validated against the permanent histology in both healthy and diseased tissue [20–23].

Regarding its use for real-time intraoperative margin assessment while the primary excision procedure is in progress, the rationale arises from the effort and time required to process and interpret specimens using histology, as well as from the limitations of other current intraoperative methods [6, 33]. For frozen section and imprint cytology margin assessment, due to the additional time (estimated by one group to be approximately 30 min) and inflexible staffing requirement of having a specially-trained pathologist in proximity to the operating room, institutional and surgeon uptake has been poor [6, 33]. OCT is attractive as an alternative as it can be completed rapidly, can scan a large specimen surface area within a timeframe compatible with surgery, and has the potential to be interpreted by the surgeon after training. Preclinical and clinical studies have thus far bolstered the contention that OCT may provide a high degree of accuracy compared to histology and other intraoperative imaging technologies presently in use, such as radiography and ultrasound [6, 33]. Previous work has demonstrated comparability to histology in both intraoperative and postoperative settings [17, 20–32, 37–40]. More recently, results from four studies related to the WF-OCT system evaluated in this report have been published, including three studies in human breast tissue and one in tissues of the head and neck [20–23].

The present report builds on these prior studies by demonstrating that normal and abnormal tissue from organs other than the breast may be successfully imaged and correlated to permanent histology by the OTIS 2.0 system. Although this study was performed using tissue obtained post-mortem, we do not currently see a use for this technology in a post-mortem setting. The study was performed here because it gave us access to multiple tissue specimens and organs which would have been difficult to obtain through normal, standard-of-care pathology processing.

Our discussion focuses on the use of this technology in breast and head-and-neck tissue types, because currently these represent the biggest unmet need in intraoperative specimen analysis and is where the largest body of work in this field has been performed. There is definitely a need in other tissue types and many of them leverage intraoperative frozen sectioning. Breast conserving surgery seldom uses frozen sectioning as it is very difficult to freeze breast tissue, creating the larger need in those types of surgeries.

We acknowledge several limitations to this study. First, the study’s generalizability is limited by its design, with a small number of evaluators, and using *postmortem* tissues from a single donor with ongoing disease processes at autopsy. Although a single-subject design that contains both normal and abnormal pathology provides some unique benefits, further study of fresh, non-diseased tissues from multiple donors and with additional readers is warranted to confirm that correlation of histology and WF-OCT remains consistent and robust across other settings. Second, the effect of WF-OCT imaging and analysis on timing and convenience of operative workflow was not assessed in this study, though others have found the impact to be minimal [20–23]. As the time required to scan and interpret images is not immaterial, this is an important metric for all future studies of intraoperative utility. Lastly, although the WF-OCT system evaluated in this study is FDA-cleared in the United States and available worldwide, no clinical trial has yet investigated its efficacy in reducing reoperations for positive margins. A prospective multi-center, randomized, controlled study to investigate this question in breast conserving surgery is currently under way. Considering the above limitations, the results presented here add to the body of evidence that WF-OCT imaging using the system can be used to identify and characterize the microarchitecture of normal and pathological tissue from a variety of human organ types.

In closing, in this autopsy-based study, both a trained pathologist and a clinical scientist were able to use WF-OCT images to identify key normal and pathological features and layers across a variety of tissue types using a single, FDA-cleared device, with direct correlation to the same features and layers observed by standard-of-care histology. Although limited to a single subject, these results are foundational in enabling the design and execution of larger clinical trials to assess if WF-OCT has the potential to bridge the gap between the immediate information needs of the operating room and the time delay inherent to tissue processing and pathology. Further clinical study of the utility of WF-OCT imaging for margin assessment at the time of surgical resection is warranted.

## Data Availability

The raw data supporting the conclusion of this article will be made available by the authors, without undue reservation.

## Abbreviations

BCS, breast-conserving surgery; FDA, food and drug administration; OCT, optical coherence tomography; WF-OCT, wide-field optical coherence tomography.

## References

[1] American Cancer Society. Cancer facts & Figures 2021 (2021). Available from: https://www.cancer.org/research/cancer-facts-statistics/all-cancer-facts-figures/cancer-facts-figures-2022.html (Accessed January 4, 2023).

[2] Miller KD Nogueira L Mariotto AB Rowland JH Yabroff KR Alfano CM Cancer treatment and survivorship statistics, 2019. CA Cancer J Clin (2019) 69:363–85. 10.3322/caac.21565 31184787

[3] Clarke M Collins R Darby S Davies C Elphinstone P Evans V Effects of radiotherapy and of differences in the extent of surgery for early breast cancer on local recurrence and 15-year survival: An overview of the randomised trials. Lancet (2005) 366:2087–106. 10.1016/S0140-6736(05)67887-7 16360786

[4] Moran MS Schnitt SJ Giuliano AE Harris JR Khan SA Horton J Society of Surgical Oncology-American Society for Radiation Oncology consensus guideline on margins for breast-conserving surgery with whole-breast irradiation in stages I and II invasive breast cancer. Ann Surg Oncol (2014) 21:704–16. 10.1245/s10434-014-3481-4 24515565

[5] Morrow M Van Zee KJ Solin LJ Houssami N Chavez-MacGregor M Harris JR Society of surgical oncology-American society for radiation oncology-American society of clinical oncology consensus guideline on margins for breast-conserving surgery with whole-breast irradiation in ductal carcinoma *in situ* . Ann Surg Oncol (2016) 23:3801–10. 10.1245/s10434-016-5449-z 27527714 PMC5047939

[6] Voskuil FJ Vonk J van der Vegt B Kruijff S Ntziachristos V van der Zaag PJ Intraoperative imaging in pathology-assisted surgery. Nat Biomed Eng (2022) 6:503–14. 10.1038/s41551-021-00808-8 34750537

[7] Schnitt SJ Moran MS Giuliano AE. Lumpectomy margins for invasive breast cancer and ductal carcinoma *in situ*: Current guideline recommendations, their implications, and impact. J Clin Oncol (2020) 38:2240–5. 10.1200/JCO.19.03213 32442067

[8] Atkins J Al Mushawah F Appleton CM Cyr AE Gillanders WE Aft RL Positive margin rates following breast-conserving surgery for stage I-iii breast cancer: Palpable versus nonpalpable tumors. J Surg Res (2012) 177:109–15. 10.1016/j.jss.2012.03.045 22516344 PMC3924771

[9] Balch GC Mithani SK Simpson JF Kelley MC. Accuracy of intraoperative gross examination of surgical margin status in women undergoing partial mastectomy for breast malignancy. Am Surg (2005) 71:22–8. 10.1177/000313480507100104 15757052

[10] Brown JQ Bydlon TM Richards LM Yu B Kennedy SA Geradts J Optical assessment of tumor resection margins in the breast. IEEE J Sel Top Quan Electron (2010) 16:530–44. 10.1109/jstqe.2009.2033257 PMC3085495 21544237

[11] Fleming FJ Hill AD Mc Dermott EW O’Doherty A O’Higgins NJ Quinn CM. Intraoperative margin assessment and re-excision rate in breast conserving surgery. Eur J Surg Oncol (2004) 30:233–7. 10.1016/j.ejso.2003.11.008 15028301

[12] McCahill LE Single RM Aiello Bowles EJ Feigelson HS James TA Barney T Variability in reexcision following breast conservation surgery. JAMA (2012) 307:467–75. 10.1001/jama.2012.43 22298678

[13] Bolshinsky V Lin MJ Serpell J Leung M Wolfe R McLean C Frequency of residual melanoma in wide local excision (WLE) specimens after complete excisional biopsy. J Am Acad Dermatol (2016) 74:102–7. 10.1016/j.jaad.2015.08.065 26601566

[14] Drexler W Fujimoto JG. Optical coherence tomography: Technology and applications. Switzerland AG: Springer International Publishing (2015).

[15] Huang D Swanson EA Lin CP Schuman JS Stinson WG Chang W Optical coherence tomography. Science (1991) 254:1178–81. 10.1126/science.1957169 1957169 PMC4638169

[16] Subban V Raffel OC. Optical coherence tomography: Fundamentals and clinical utility. Cardiovasc Diagn Ther (2020) 10:1389–414. 10.21037/cdt-20-253 33224764 PMC7666937

[17] Wang J Xu Y Boppart SA. Review of optical coherence tomography in oncology. J Biomed Opt (2017) 22:1–23. 10.1117/1.JBO.22.12.121711 PMC5741100 29274145

[18] Zysk AM Nguyen FT Oldenburg AL Marks DL Boppart SA. Optical coherence tomography: A review of clinical development from bench to bedside. J Biomed Opt (2007) 12:051403. 10.1117/1.2793736 17994864

[19] Tung ET Yim KHC Li CL Cheung CY Chan YC. Optical coherence tomography in peripheral arterial disease: A systematic review. Int J Clin Pract (2021) 75:e14628. 10.1111/ijcp.14628 34258814

[20] Badhey AK Schwarz JS Laitman BM Veremis BM Westra WH Yao M Intraoperative use of wide-field optical coherence tomography to evaluate tissue microstructure in the oral cavity and oropharynx. JAMA Otolaryngol Head Neck Surg (2022) 149:71–8. 10.1001/jamaoto.2022.3763 PMC9856682 36454583

[21] DuPree BB Papez MJ Pirruccello E Hassenflug A. Potential utility of adjunct imaging with wide‐field optical coherence tomography for gross and microscopic evaluation of breast specimens in real‐time in the operating suite. Indian J Surg (2021) 84:751–6. 10.1007/s12262-021-03079-4

[22] Schmidt H Connolly C Jaffer S Oza T Weltz CR Port ER Evaluation of surgically excised breast tissue microstructure using wide-field optical coherence tomography. Breast J (2020) 26:917–23. 10.1111/tbj.13663 31612563

[23] Ha R Friedlander LC Hibshoosh H Hendon C Feldman S Ahn S Optical coherence tomography: A novel imaging method for post-lumpectomy breast margin assessment-a multi-reader study. Acad Radiol (2018) 25:279–87. 10.1016/j.acra.2017.09.018 29174226

[24] Erickson-Bhatt SJ Nolan RM Shemonski ND Adie SG Putney J Darga D Real-time imaging of the resection bed using a handheld probe to reduce incidence of microscopic positive margins in cancer surgery. Cancer Res (2015) 75:3706–12. 10.1158/0008-5472.CAN-15-0464 26374464 PMC4749141

[25] Conti de Freitas LC Phelan E Liu L Gardecki J Namati E Warger WC Optical coherence tomography imaging during thyroid and parathyroid surgery: A novel system of tissue identification and differentiation to obviate tissue resection and frozen section. Head Neck (2014) 36:1329–34. 10.1002/hed.23452 23956009 PMC5777931

[26] Gan Y Tsay D Amir SB Marboe CC Hendon CP. Automated classification of optical coherence tomography images of human atrial tissue. J Biomed Opt (2016) 21:101407. 10.1117/1.JBO.21.10.101407 26926869 PMC5995000

[27] Gan Y Yao X Chang E Amir SB Hibshoosh H Feldman S Comparative study of texture features in OCT images at different scales for human breast tissue classification. In: 2016 38th Annual International Conference of the IEEE Engineering in Medicine and Biology Society; 16-20 August 2016; Orlando, FL, USA (2016). p. 3926–9. 10.1109/EMBC.2016.7591586 PMC6180913 28269144

[28] Hariri LP Mino-Kenudson M Lanuti M Miller AJ Mark EJ Suter MJ. Diagnosing lung carcinomas with optical coherence tomography. Ann Am Thorac Soc (2015) 12:193–201. 10.1513/AnnalsATS.201408-370OC 25562183 PMC4342833

[29] Iftimia N Cizginer S Deshpande V Pitman M Tatli S Iftimia NA Differentiation of pancreatic cysts with optical coherence tomography (OCT) imaging: An *ex vivo* pilot study. Biomed Opt Express (2011) 2:2372–82. 10.1364/BOE.2.002372 21833374 PMC3149535

[30] Jäckle S Gladkova N Feldchtein F Terentieva A Brand B Gelikonov G *In vivo* endoscopic optical coherence tomography of the human gastrointestinal tract-toward optical biopsy. Endoscopy (2000) 32:743–9. 10.1055/s-2000-7711 11068832

[31] Lee HC Zhou C Cohen DW Mondelblatt AE Wang Y Aguirre AD Integrated optical coherence tomography and optical coherence microscopy imaging of *ex vivo* human renal tissues. J Urol (2012) 187:691–9. 10.1016/j.juro.2011.09.149 22177199 PMC3366589

[32] Poneros JM Tearney GJ Shiskov M Kelsey PB Lauwers GY Nishioka NS Optical coherence tomography of the biliary tree during ERCP. Gastrointest Endosc (2002) 55:84–8. 10.1067/mge.2002.120098 11756925

[33] St John ER Al-Khudairi R Ashrafian H Athanasiou T Takats Z Hadjiminas DJ Diagnostic accuracy of intraoperative techniques for margin assessment in breast cancer surgery: A meta-analysis. Ann Surg (2017) 265:300–10. 10.1097/SLA.0000000000001897 27429028

[34] Gray RJ Pockaj BA Garvey E Blair S. Intraoperative margin management in breast-conserving surgery: A systematic review of the literature. Ann Surg Oncol (2018) 25:18–27. 10.1245/s10434-016-5756-4 28058560

[35] Schnabel F Boolbol SK Gittleman M Karni T Tafra L Feldman S A randomized prospective study of lumpectomy margin assessment with use of MarginProbe in patients with nonpalpable breast malignancies. Ann Surg Oncol (2014) 21:1589–95. 10.1245/s10434-014-3602-0 24595800 PMC3975090

[36] Ali ZA Karimi Galougahi K Maehara A Shlofmitz RA Ben-Yehuda O Mintz GS Intracoronary optical coherence tomography 2018: Current status and future directions. JACC Cardiovasc Interv (2017) 10:2473–87. 10.1016/j.jcin.2017.09.042 29268880

[37] Ang M Tan ACS Cheung CMG Keane PA Dolz-Marco R Sng CCA Optical coherence tomography angiography: A review of current and future clinical applications. Graefes Arch Clin Exp Ophthalmol (2018) 256:237–45. 10.1007/s00417-017-3896-2 29318383

[38] Fujii K Kawakami R Hirota S. Histopathological validation of optical coherence tomography findings of the coronary arteries. J Cardiol (2018) 72:179–85. 10.1016/j.jjcc.2018.03.003 29655510

[39] Vakoc BJ Fukumura D Jain RK Bouma BE. Cancer imaging by optical coherence tomography: Preclinical progress and clinical potential. Nat Rev Cancer (2012) 12:363–8. 10.1038/nrc3235 22475930 PMC3560400

[40] Nguyen FT Zysk AM Chaney EJ Kotynek JG Oliphant UJ Bellafiore FJ Intraoperative evaluation of breast tumor margins with optical coherence tomography. Cancer Res (2009) 69:8790–6. 10.1158/0008-5472.CAN-08-4340 19910294 PMC2782920

