# Supplementary material for: Wide-field optical coherence tomography for microstructural analysis of key tissue types: a proof-of-concept evaluation
Source: Pathol Oncol Res. 2023 Jul 14;29:1611167. doi: 10.3389/pore.2023.1611167 (PMC10374948; doi:10.3389/pore.2023.1611167)
Supplement: Supplementary file 3 [file DataSheet1.PDF]

SUPPLEMENTARY MATERIAL

Supplementary Figure S1: Overall Specimen Analysis Workflow. Three specimens per tissue type were collected from the donor. Using specimen ink, dots were placed on each specimen to mark orientation. Specimens were then scanned using OCT under no, low, or high vacuum pressure. After scanning, specimens were fully inked, grossed into 3 to 5 slices, and placed in individual cassettes. The cassettes were placed in formalin and transported to the biorepository lab where they were processed and slides were generated. Slides were then digitized and compared with the OCT images on a computer.

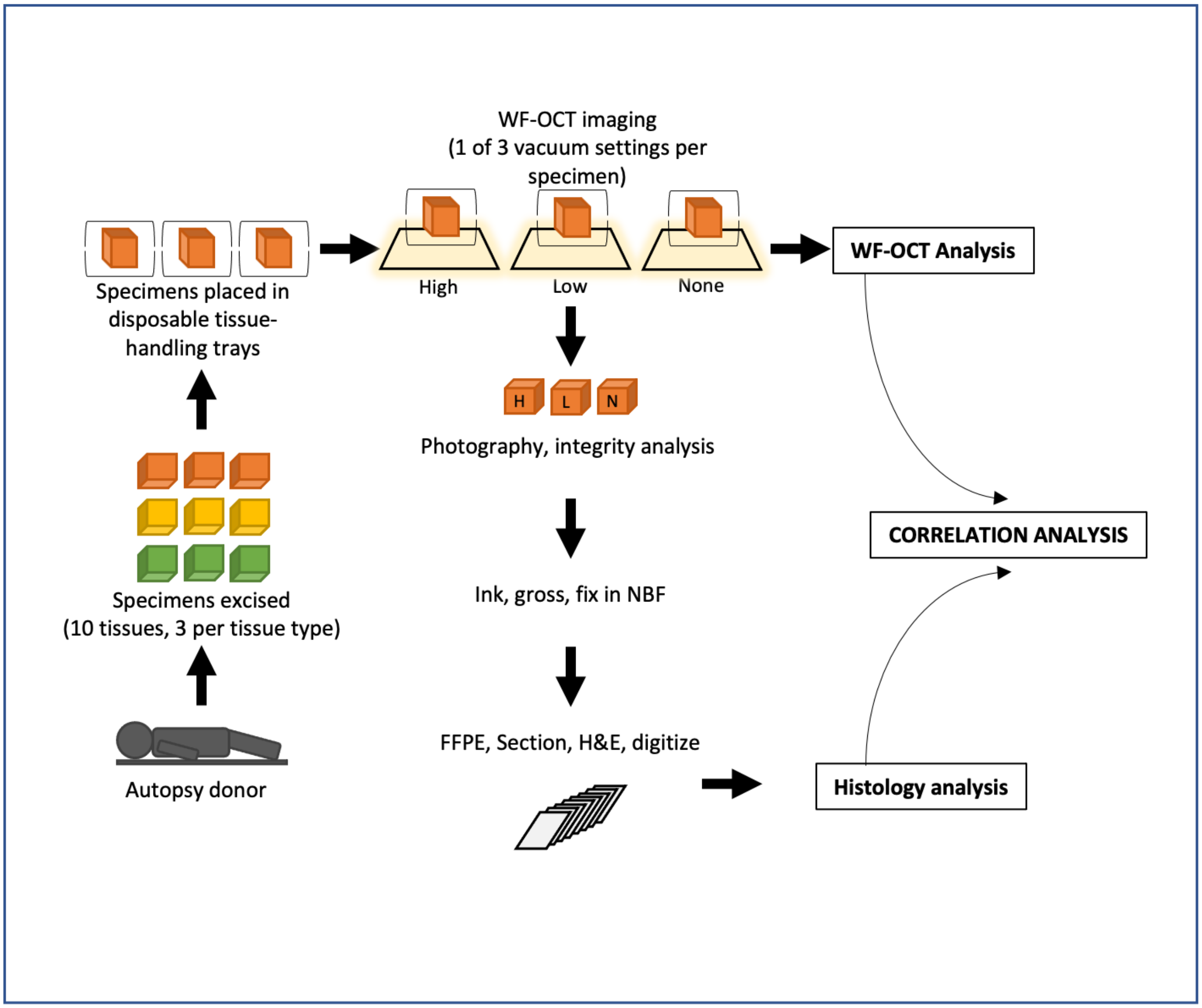

SUPPLEMENTARY VIDEOS

VIDEO1.avi	WF-OCT volumetric analysis; thyroid specimen. The system automatically processes adjacent B scans so that the user can scroll through a tissue volume (C scan).
VIDEO2.avi	WF-OCT volumetric analysis; kidney specimen. The system automatically processes adjacent B scans so that the user can scroll through a tissue volume (C scan).
VIDEO3.avi	WF-OCT volumetric analysis; liver specimen. The system automatically processes adjacent B scans so that the user can scroll through a tissue volume (C scan).

SUPPLEMENTARY INTEGRITY ANALYSIS

Specimen photographs taken before and after WF-OCT scanning were compared visually to assess the effect of vacuum pressure (non, low, or high) on specimen integrity.

Specimen integrity – 1 (Breast)

Before Scan

After Scan

1- Control

2- Low vacuum

3- Full vacuum

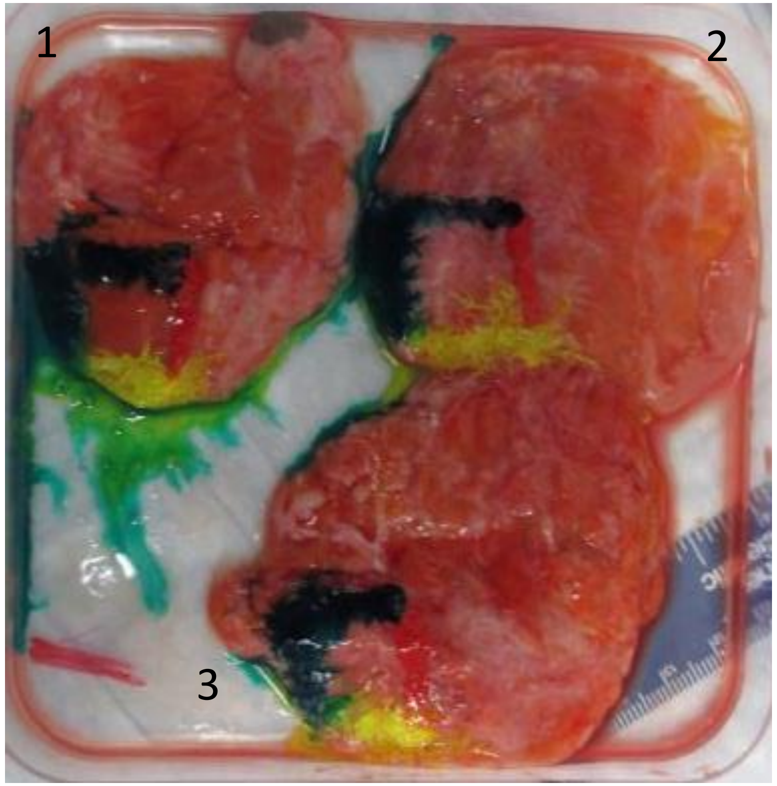

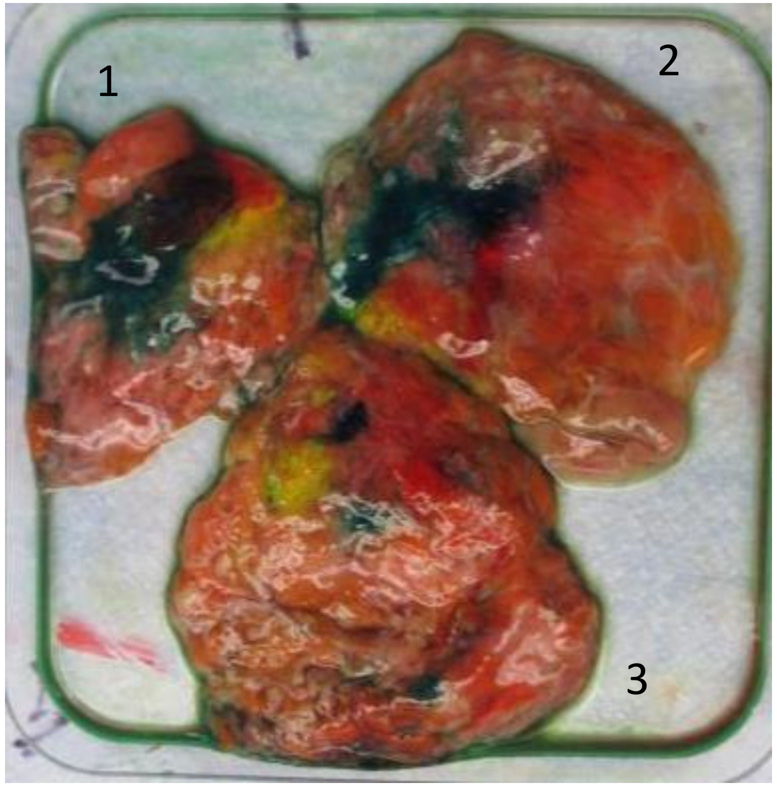

Specimen integrity – 2 (Heart)

1- Control

2- Low vacuum

3- Full vacuum

Before Scan

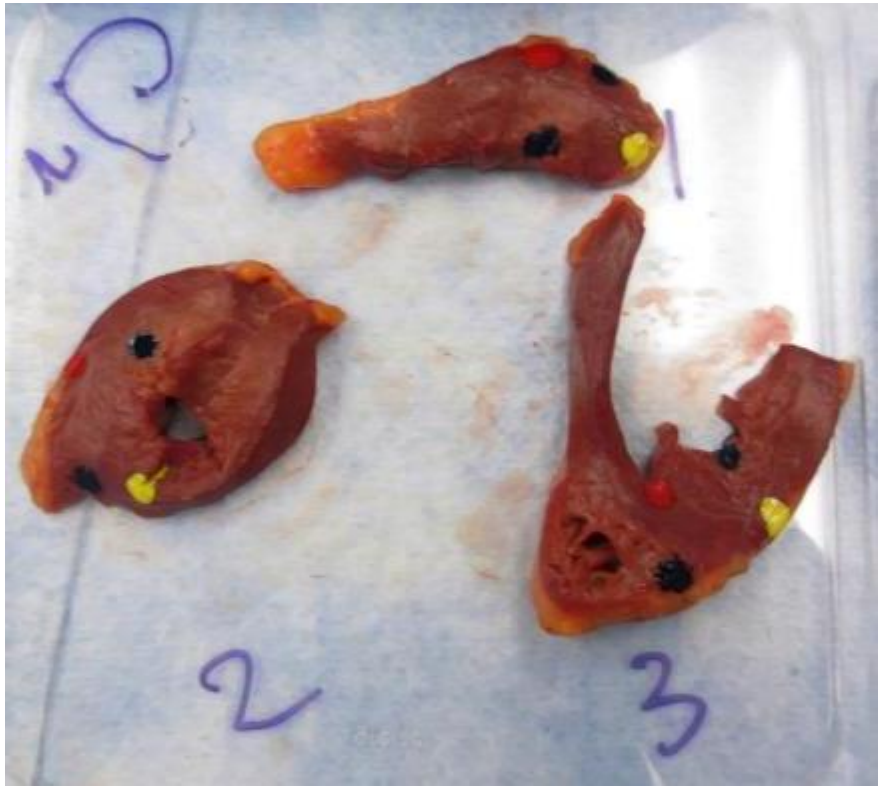

After Scan

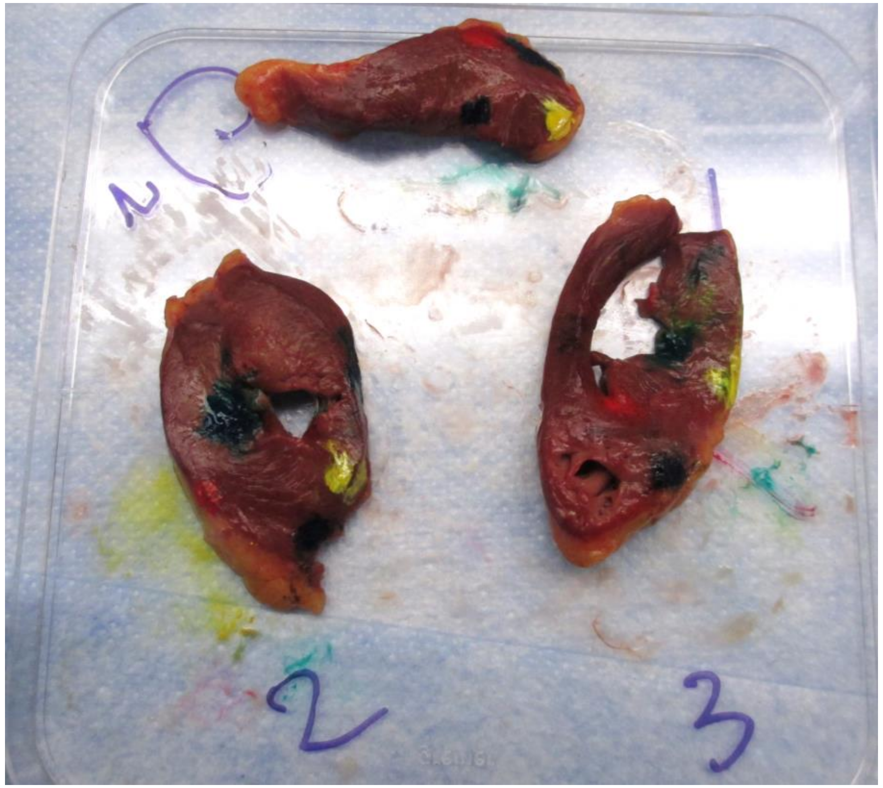

Specimen integrity – 3 (Kidney)

1- Control

2- Low vacuum

3- Full vacuum

Before Scan

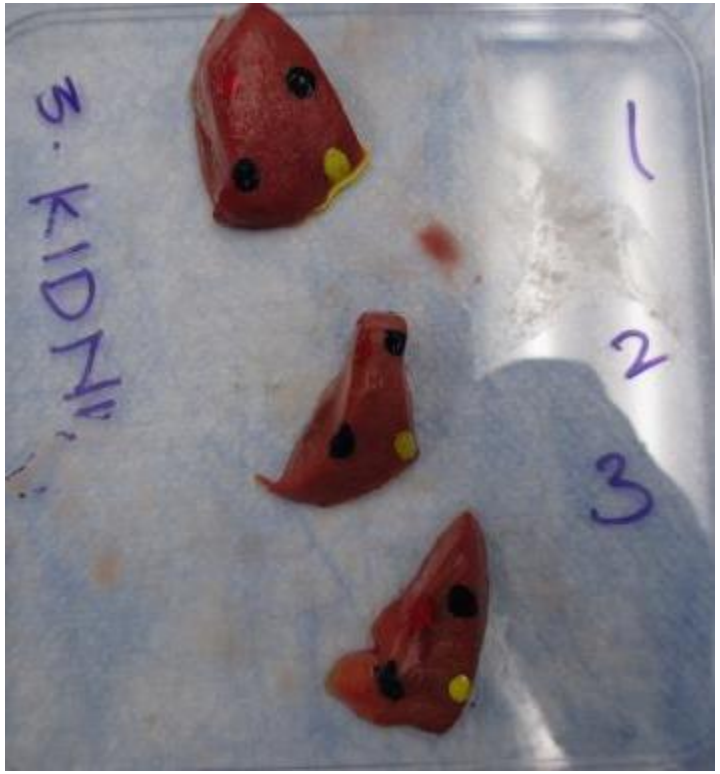

After Scan

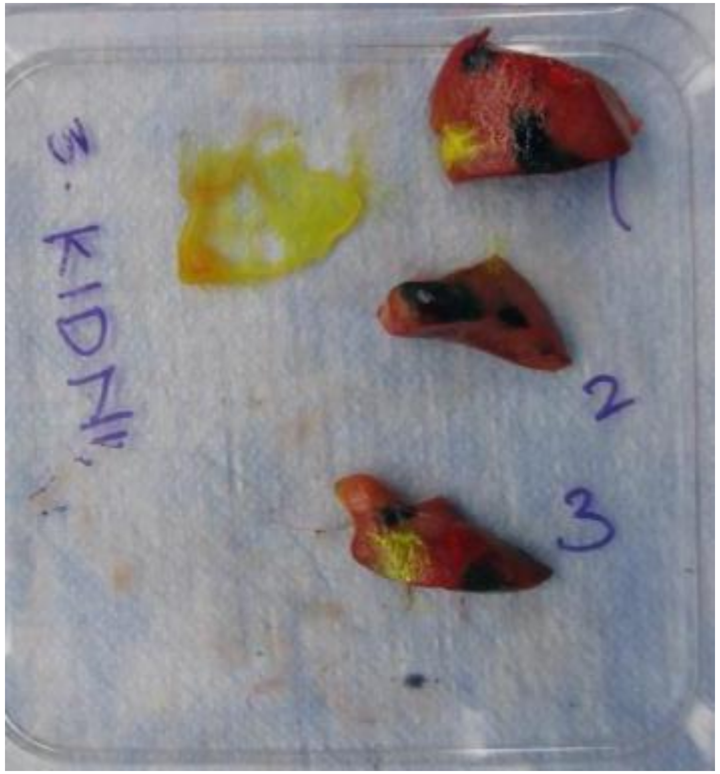

Specimen integrity – 4 (Spleen)

1- Control

2- Low vacuum

3- Full vacuum

Before Scan

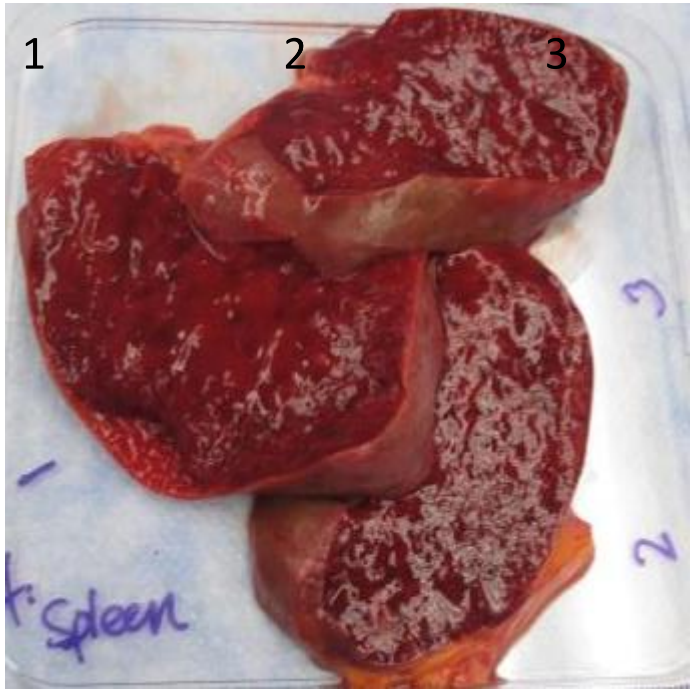

After Scan

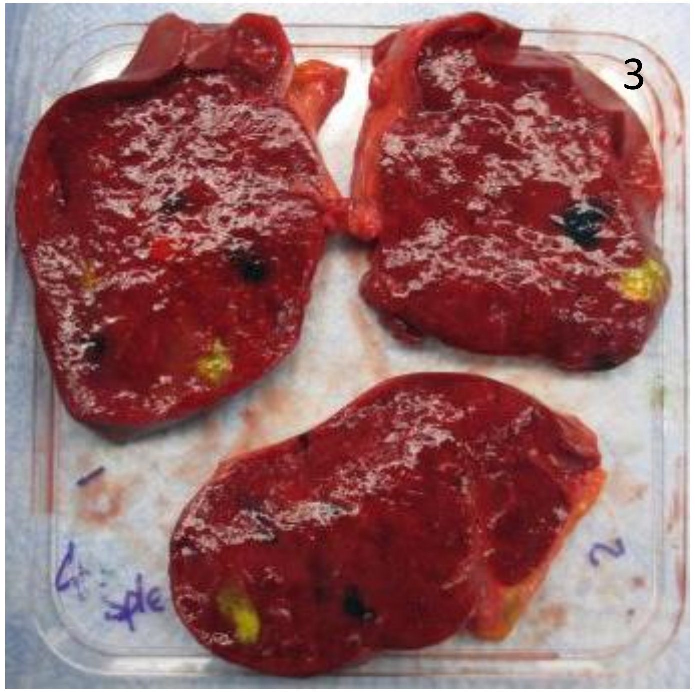

Specimen integrity – 5 (Thyroid)

Before Scan

After Scan

1- Control

2- Low vacuum

3- Full vacuum

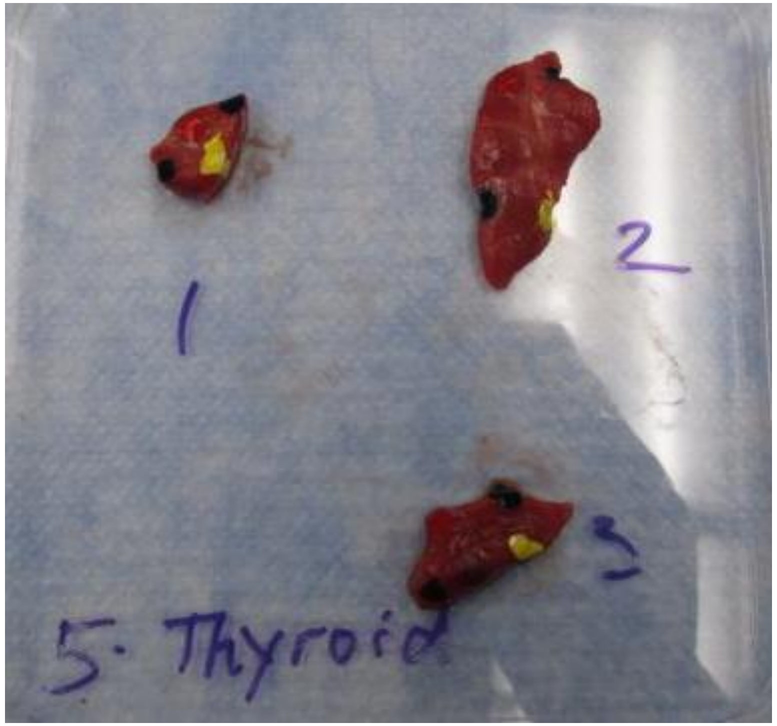

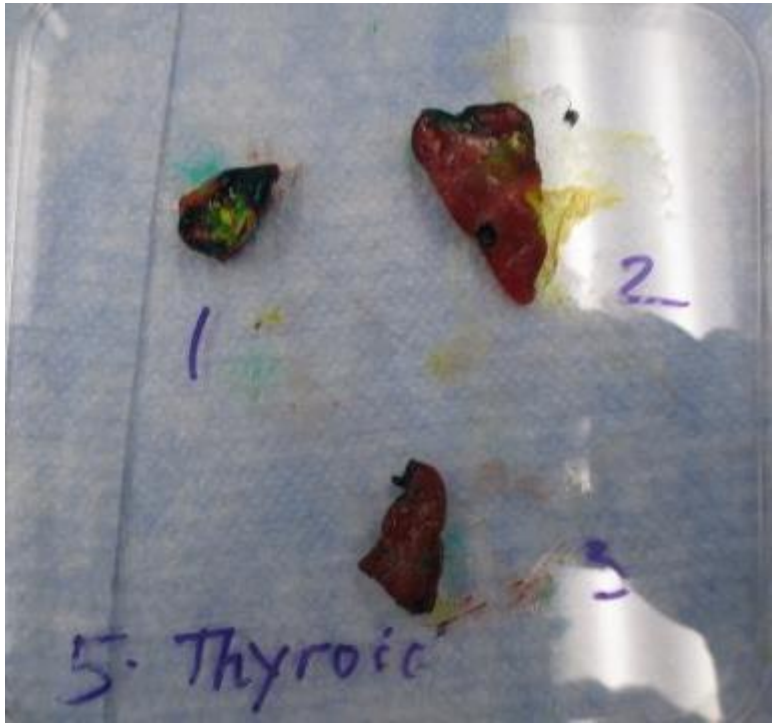

Specimen integrity – 6 (Adrenal)

Before Scan

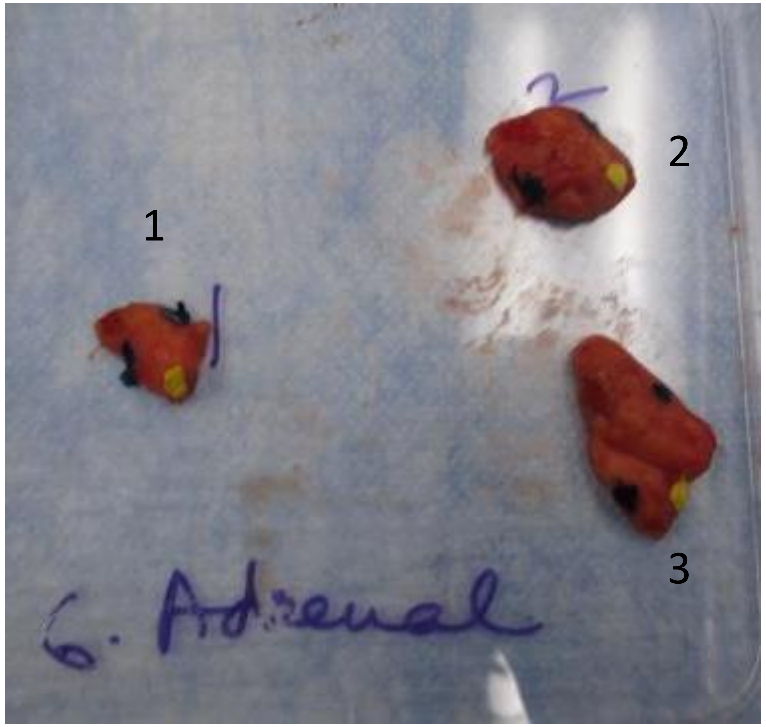

After Scan

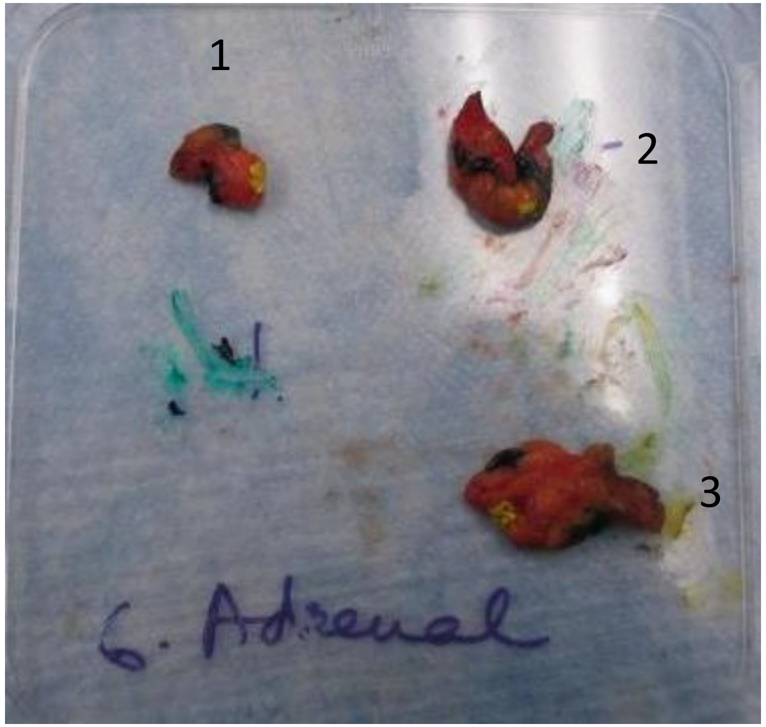

1- Control

2- Low vacuum

3- Full vacuum

Specimen integrity – 7 (Pancreas)

Before Scan

After Scan

1- Control

2- Low vacuum

3- Full vacuum

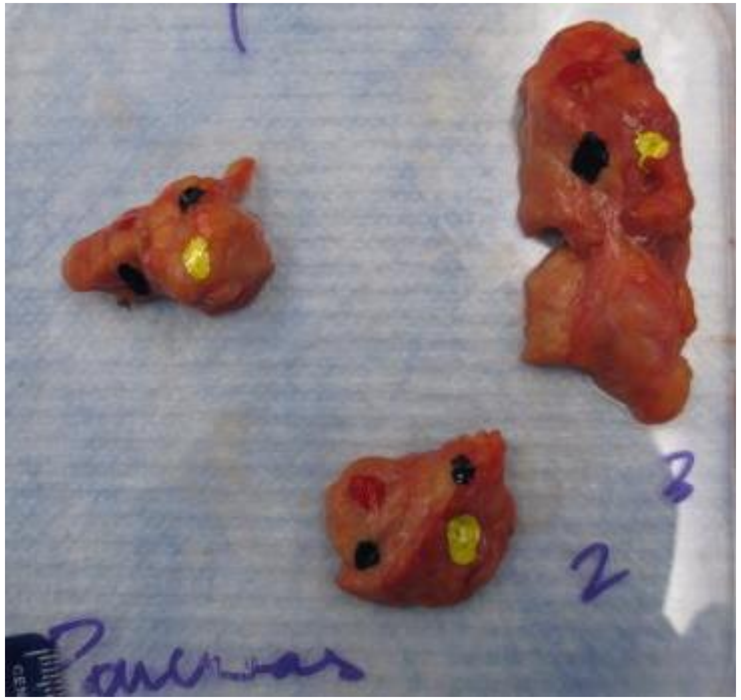

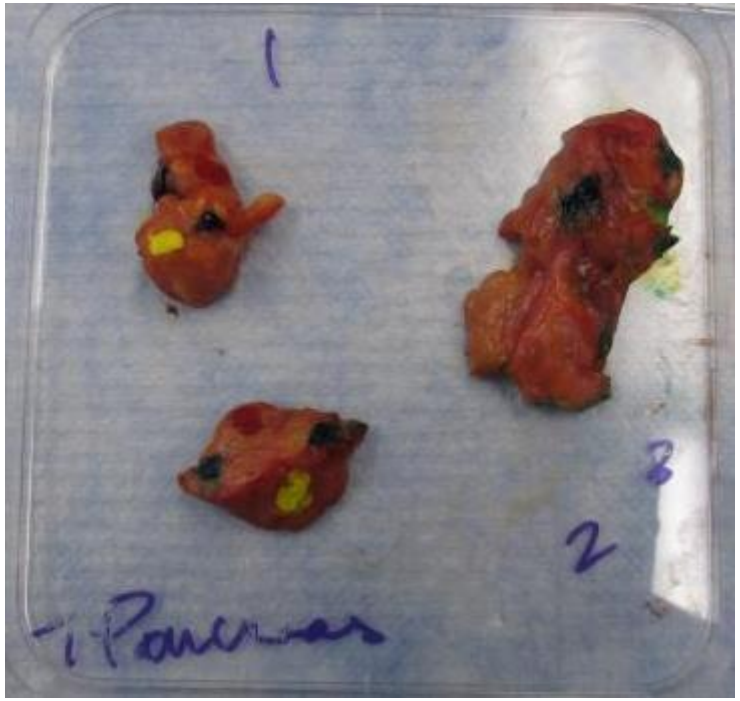

Specimen integrity – 8 (Liver)

Before Scan

After Scan

1- Control

2- Low vacuum

3- Full vacuum

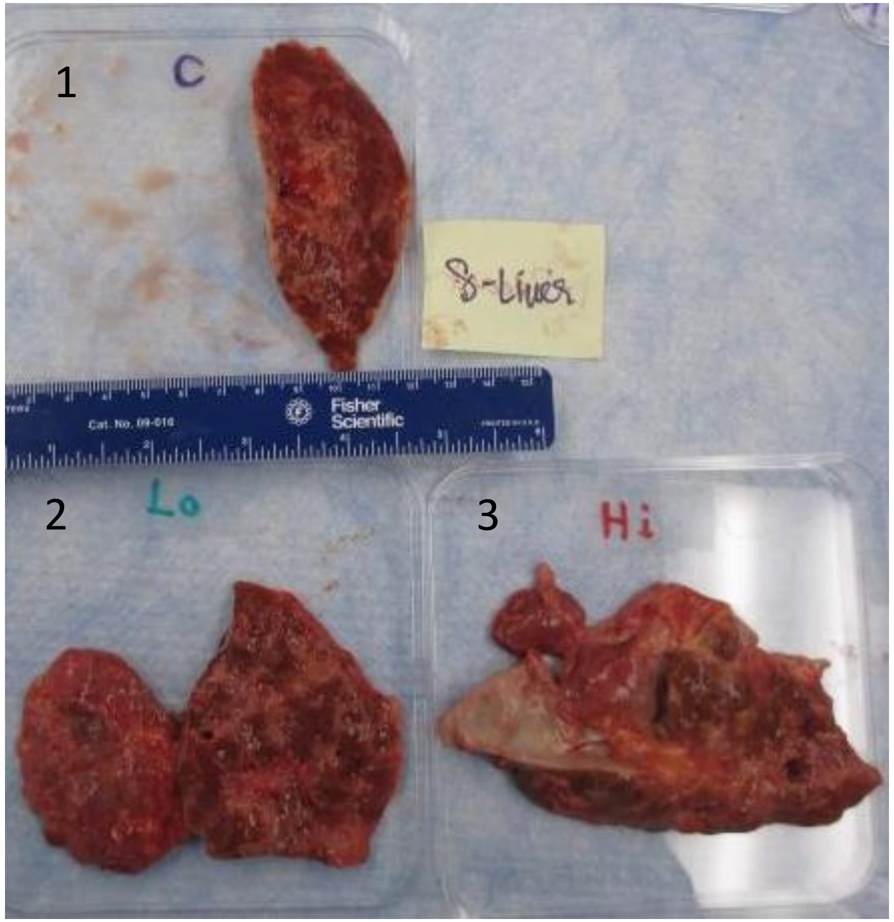

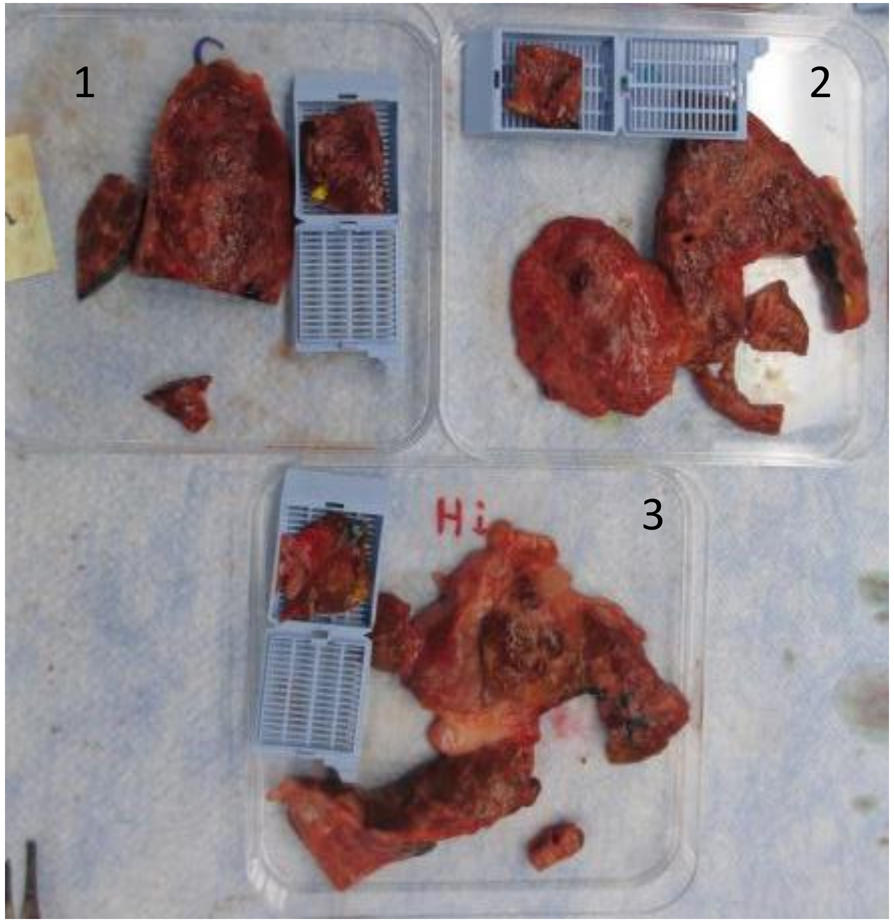

Specimen integrity – 9 (Lung)

1- Control

2- Low vacuum

3- Full vacuum

Before Scan

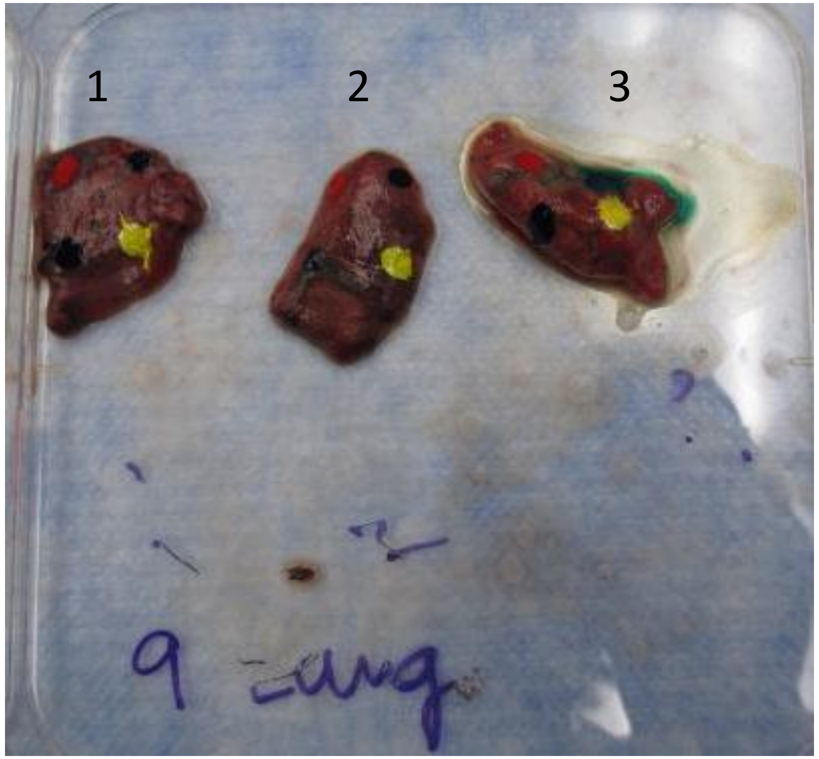

After Scan

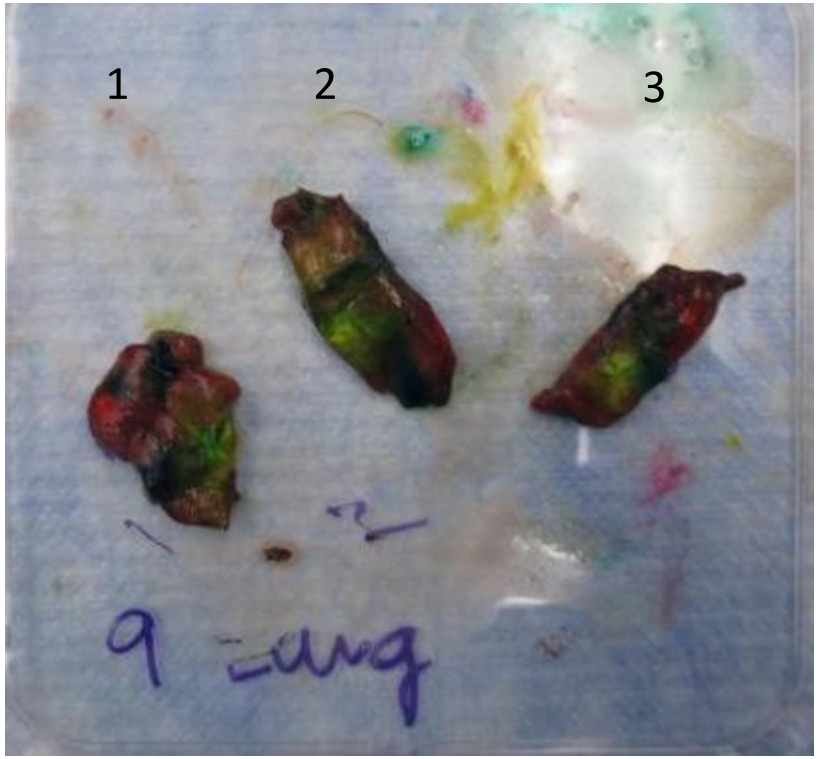

Specimen integrity – 10 (Colon)

1- Control

2- Low vacuum

3- Full vacuum

Before Scan

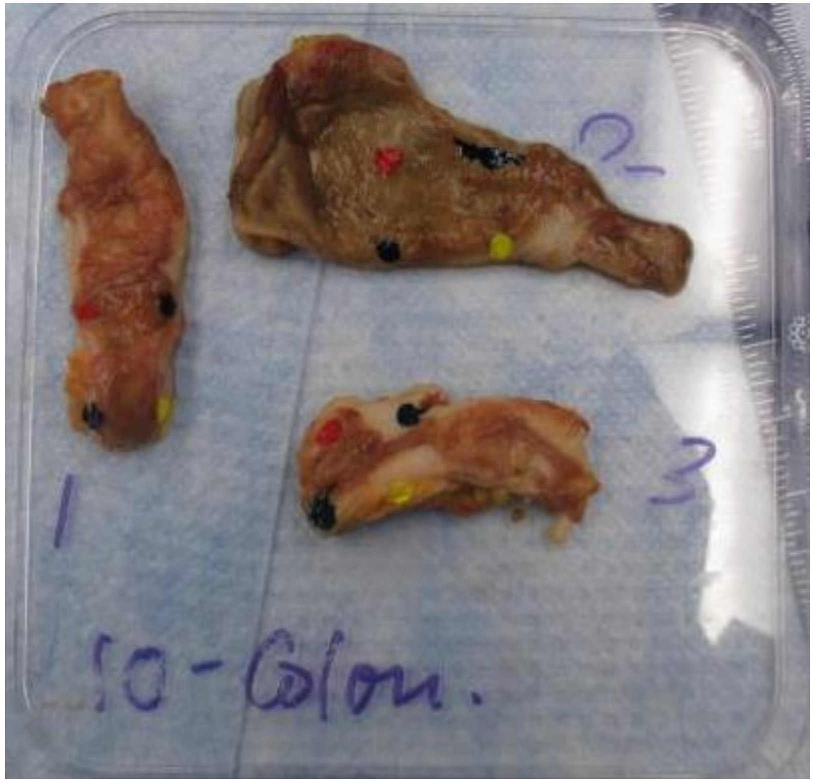

After Scan

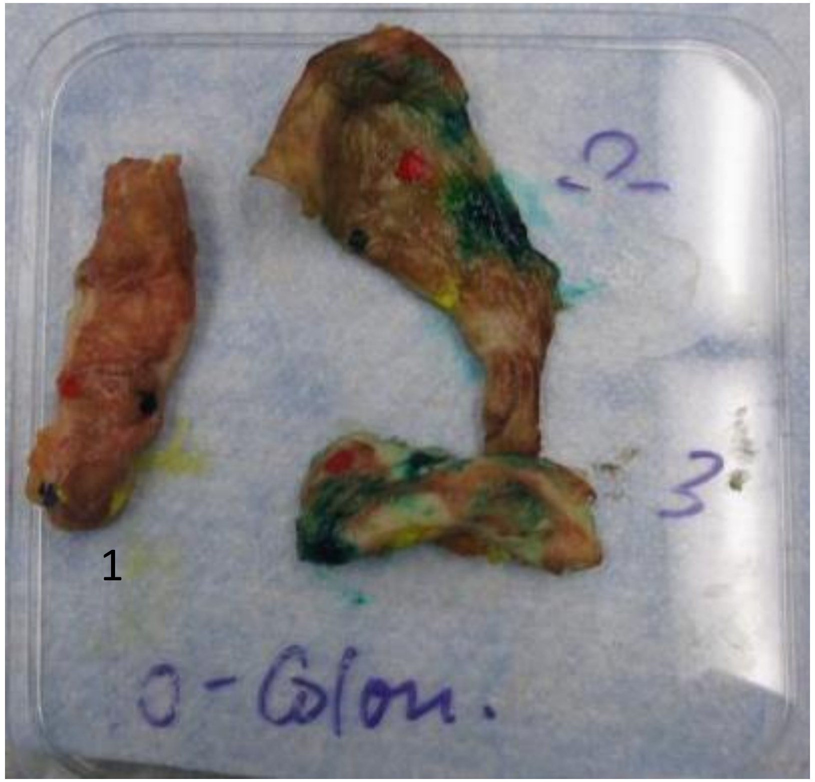

Histology slides – 1 (Breast)

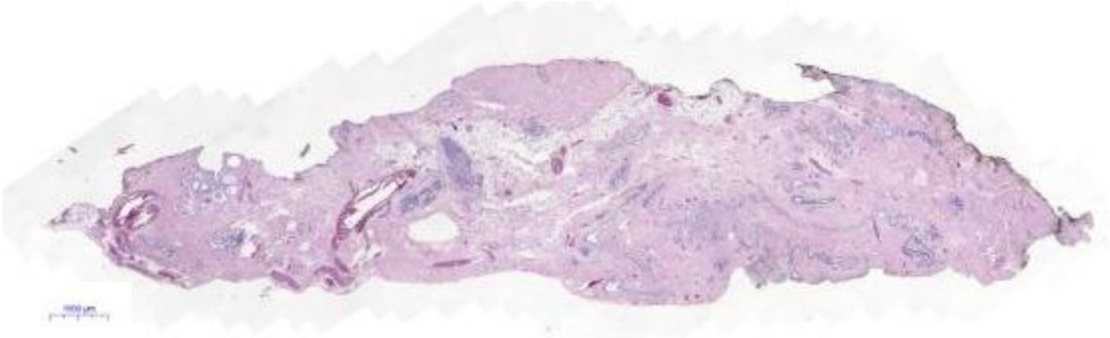

Control (1C-C)

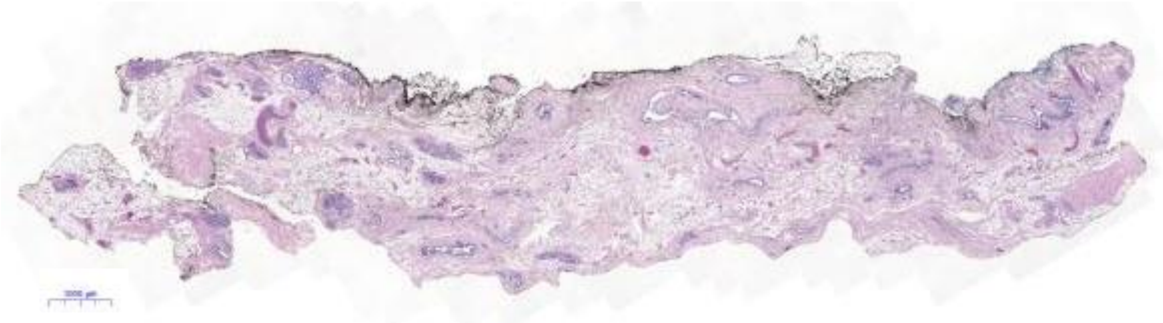

Low Vacuum (1Lo-C)

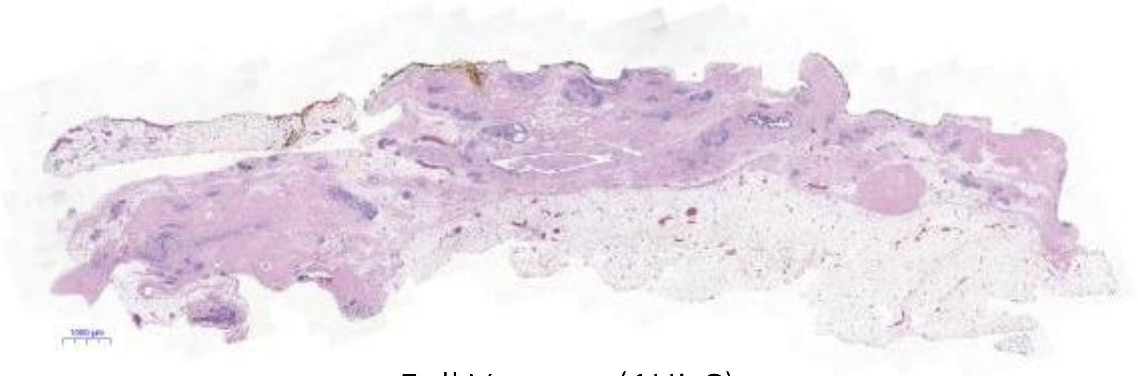

Full Vacuum (1Hi-C)

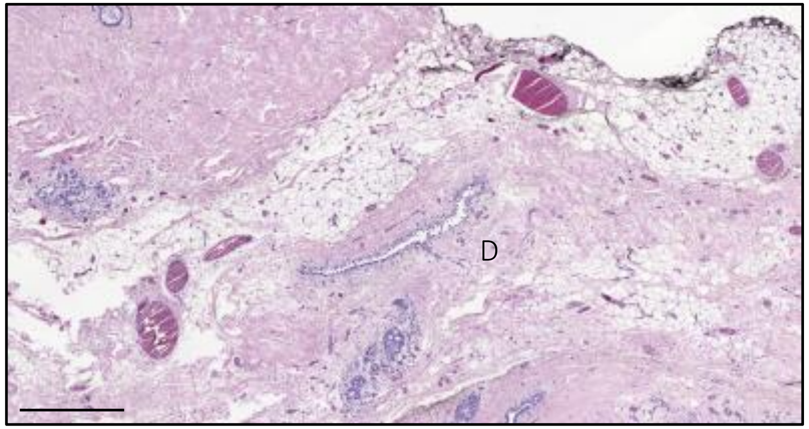

Control

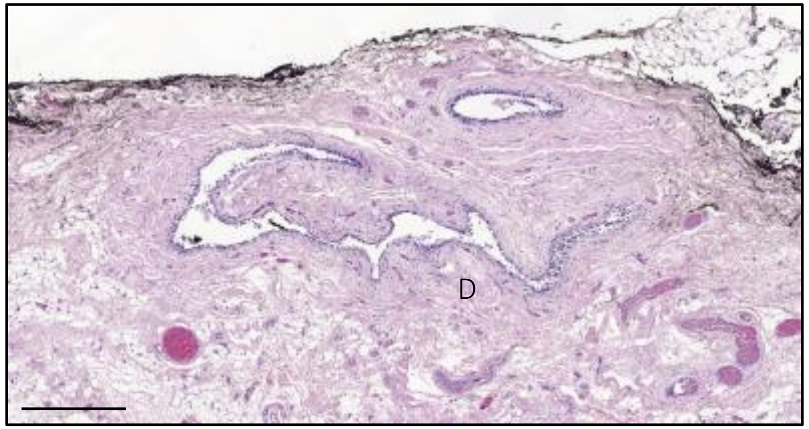

Low Vacuum

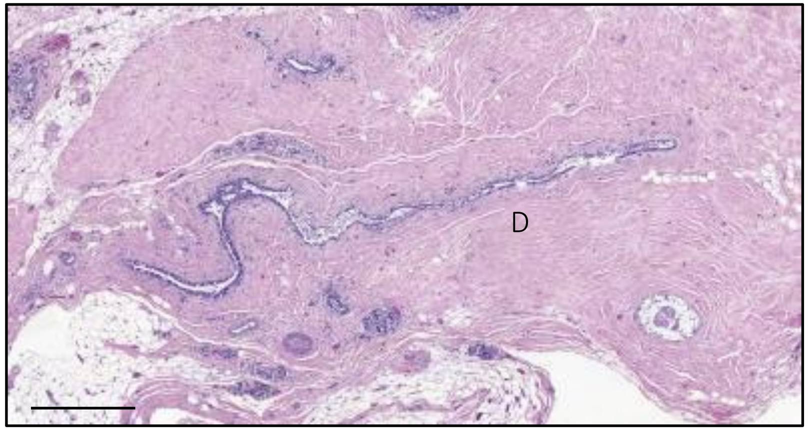

Full Vacuum

Histology slides – 2 (Heart)

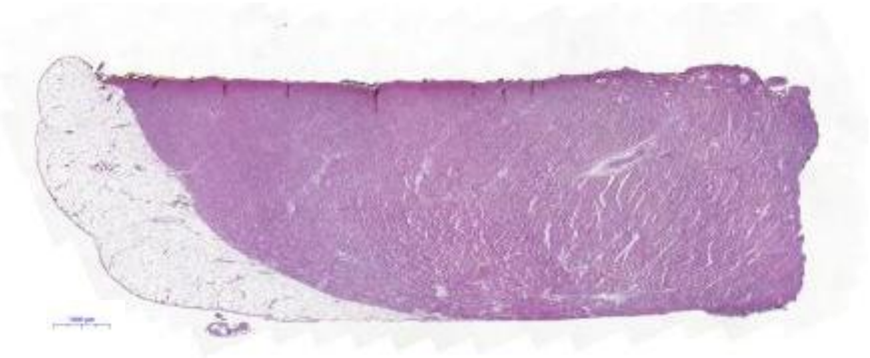

Control (2C-C)

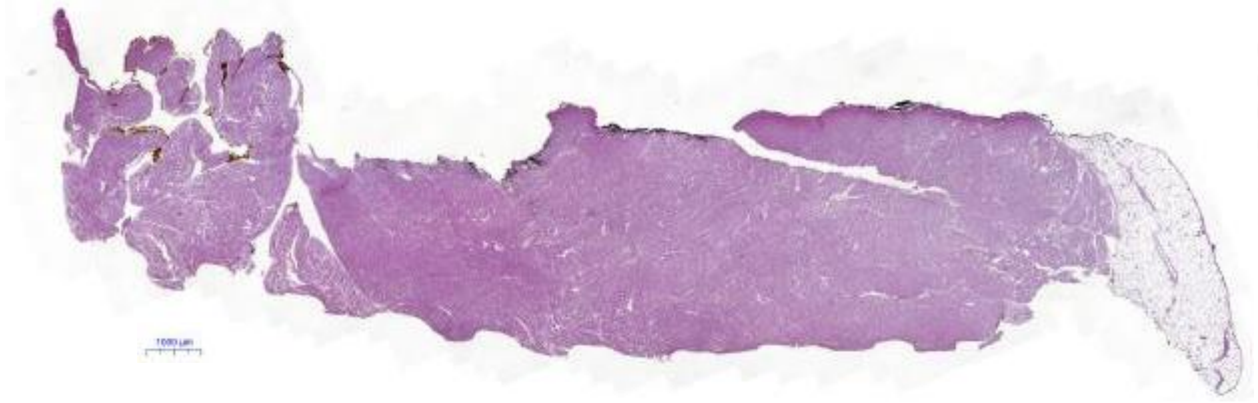

Low Vacuum (2Lo-C)

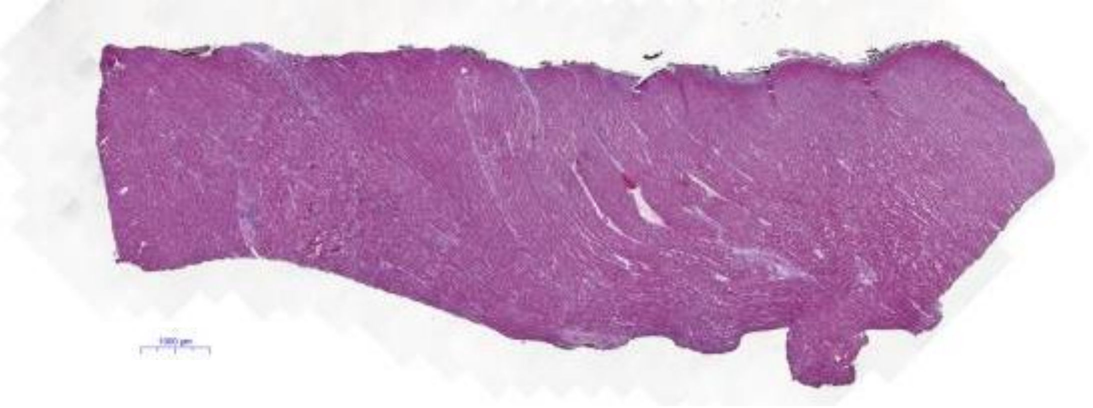

Full Vacuum (2Hi-B)

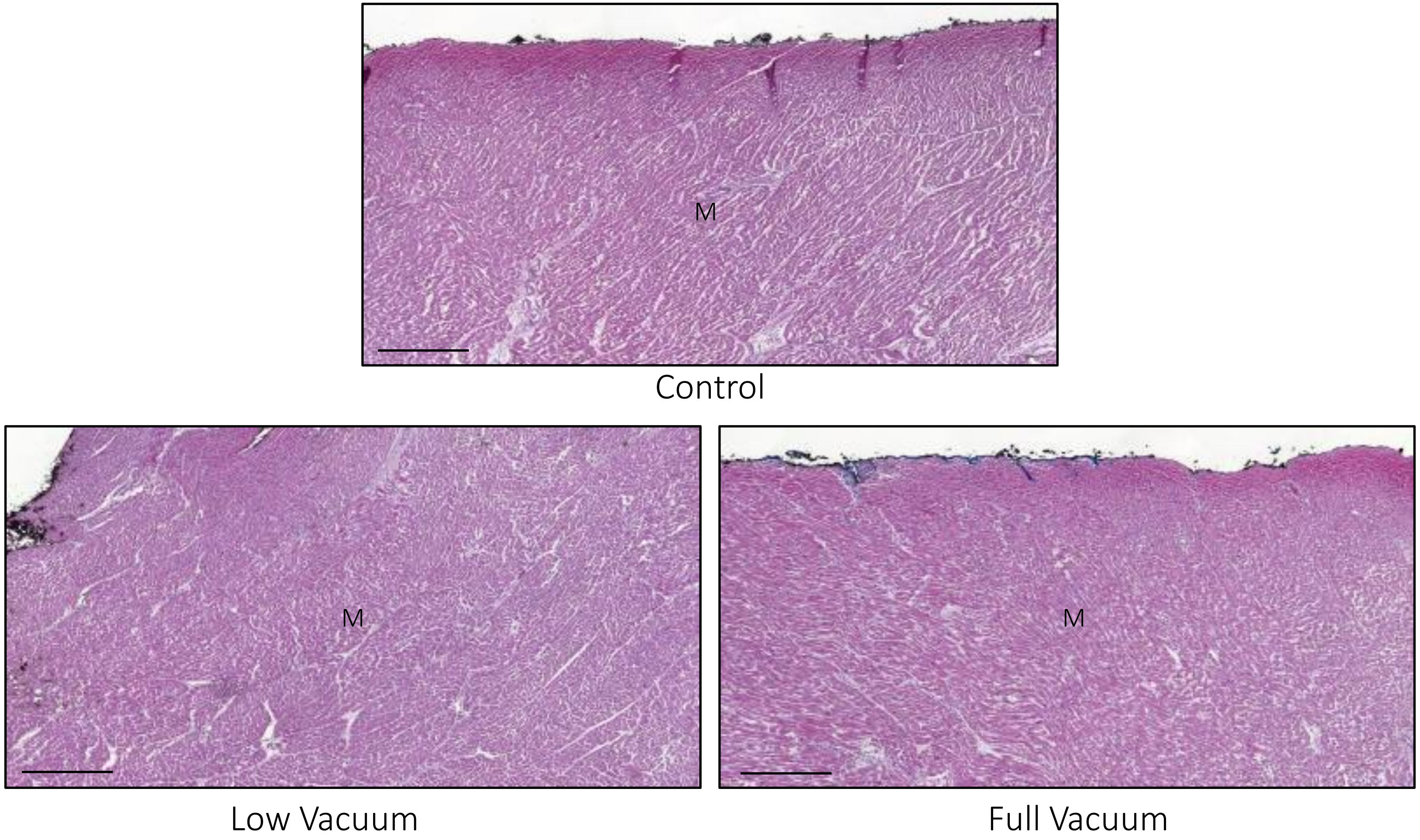
M
Control
M
Low Vacuum
M
Full Vacuum

Histology slides – 3 (Kidney)

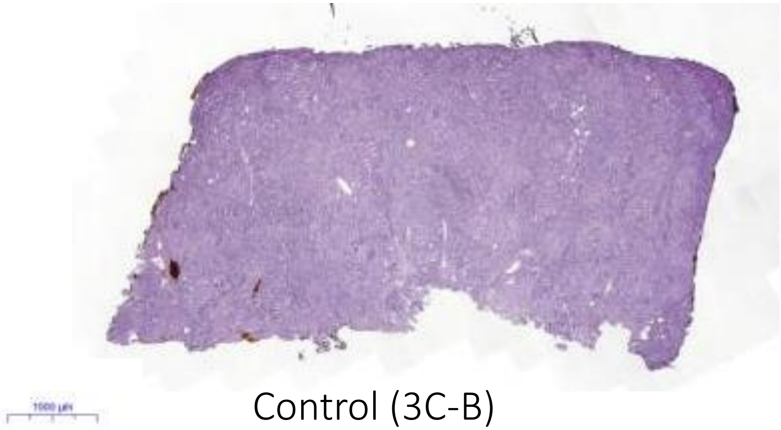

Control (3C-B)

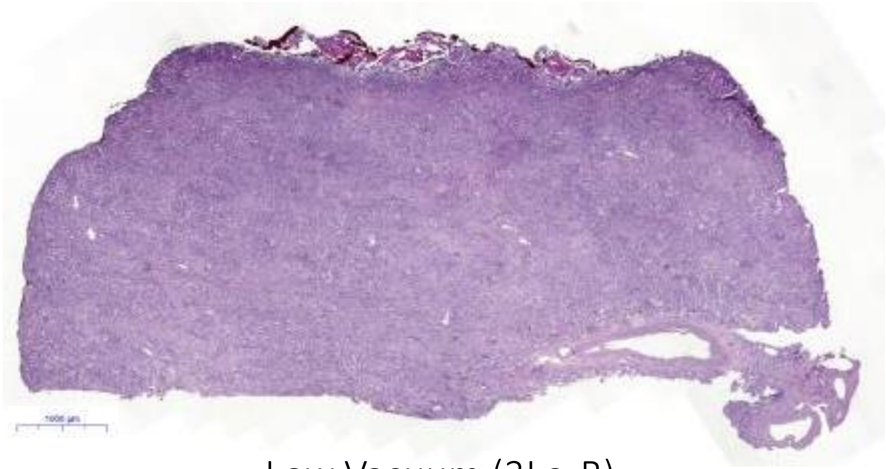

Low Vacuum (3Lo-B)

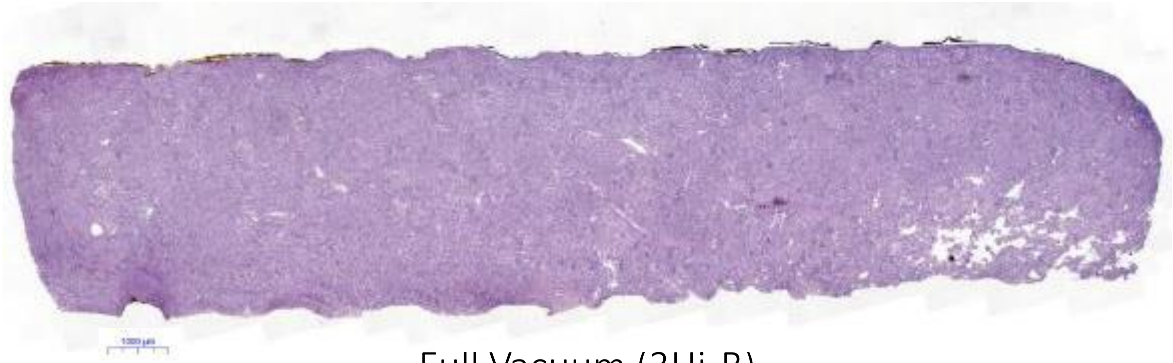

Full Vacuum (3Hi-B)

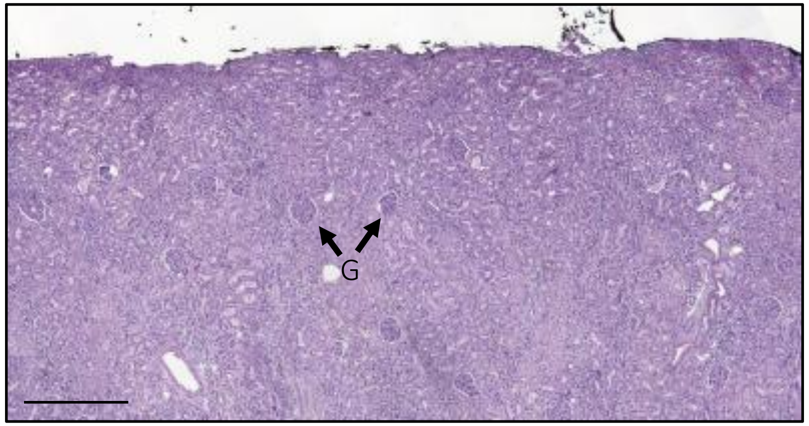

Control

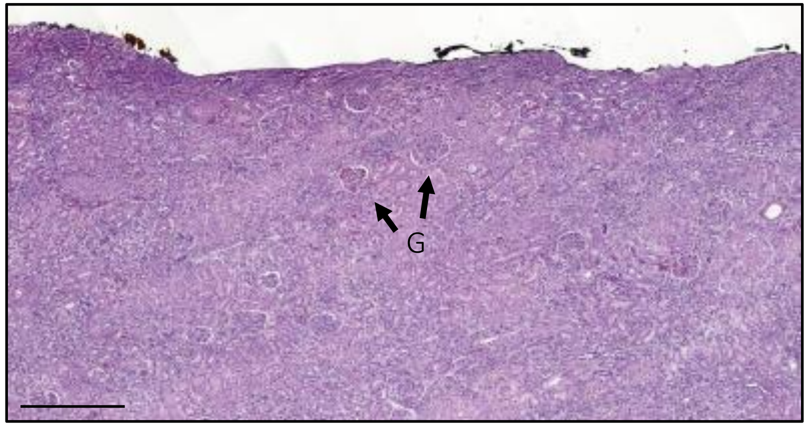

Low Vacuum

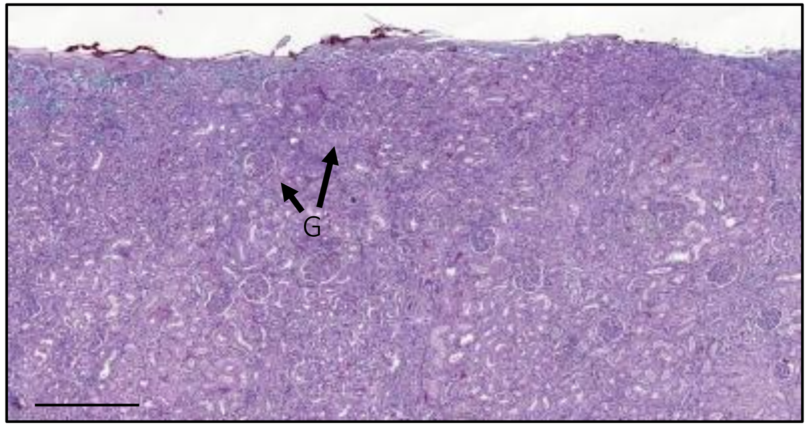

Full Vacuum

Histology slides – 4 (Spleen)

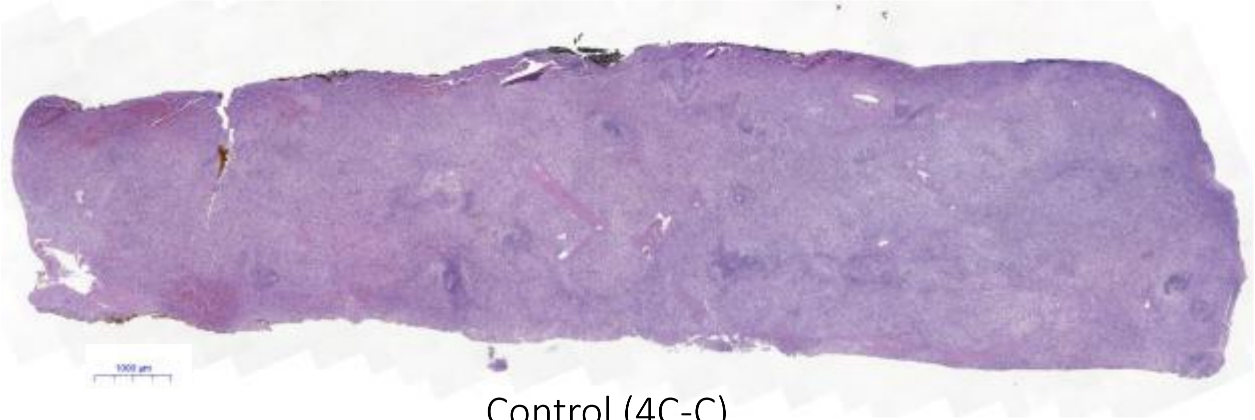

Control (4C-C)

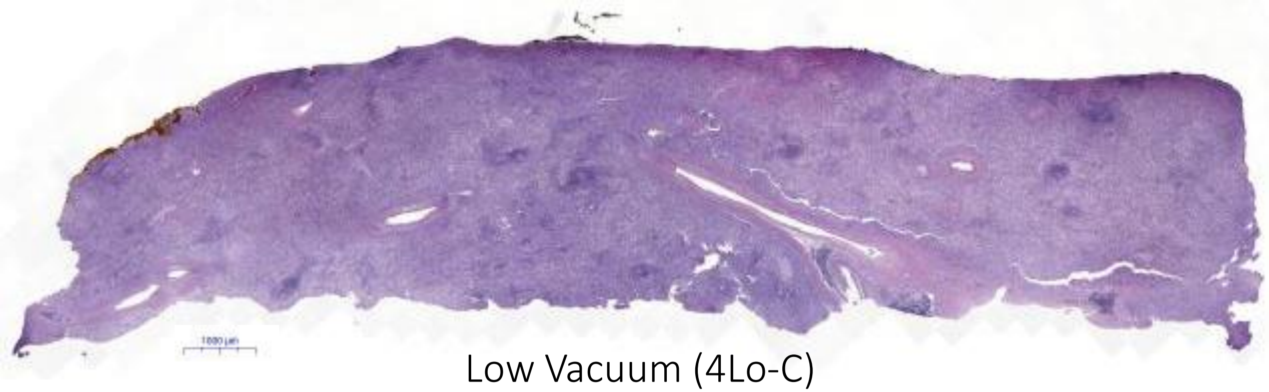

Low Vacuum (4Lo-C)

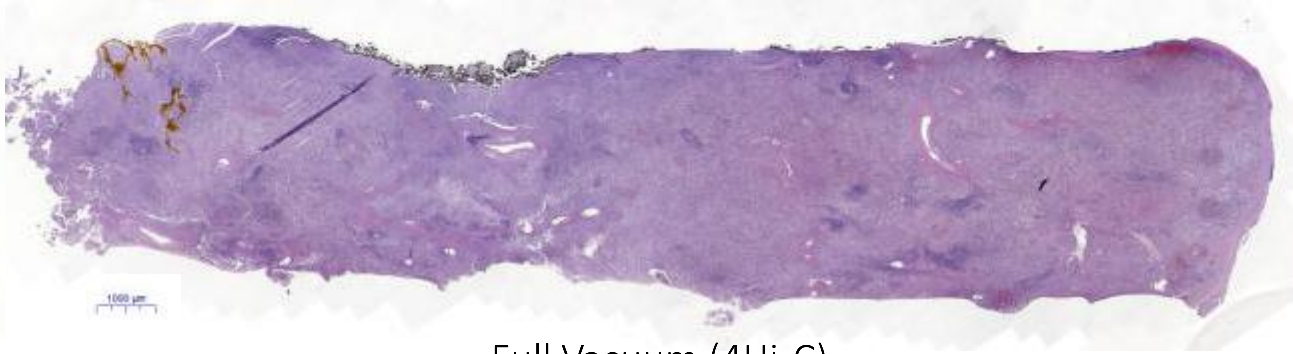

Full Vacuum (4Hi-C)

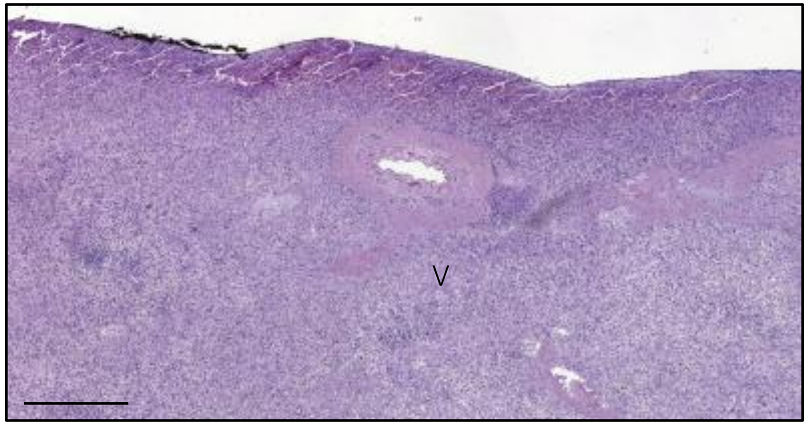

Control

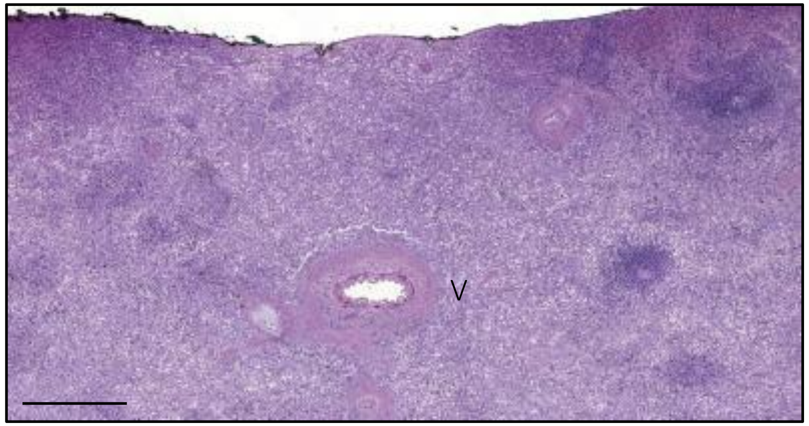

Low Vacuum

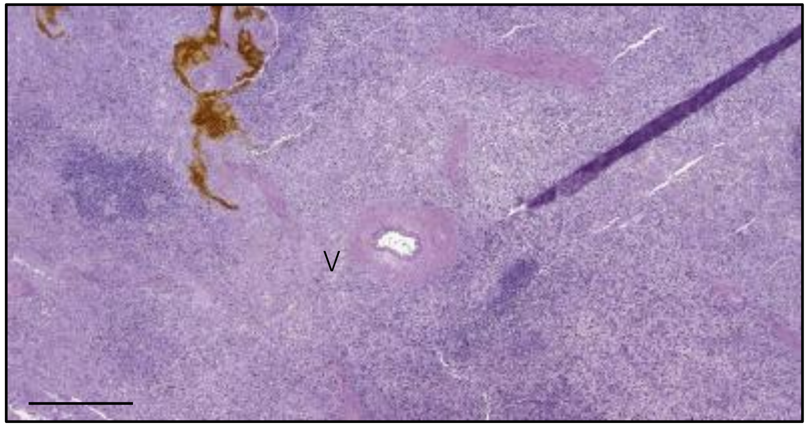

Full Vacuum

Histology slides – 5 (Thyroid)

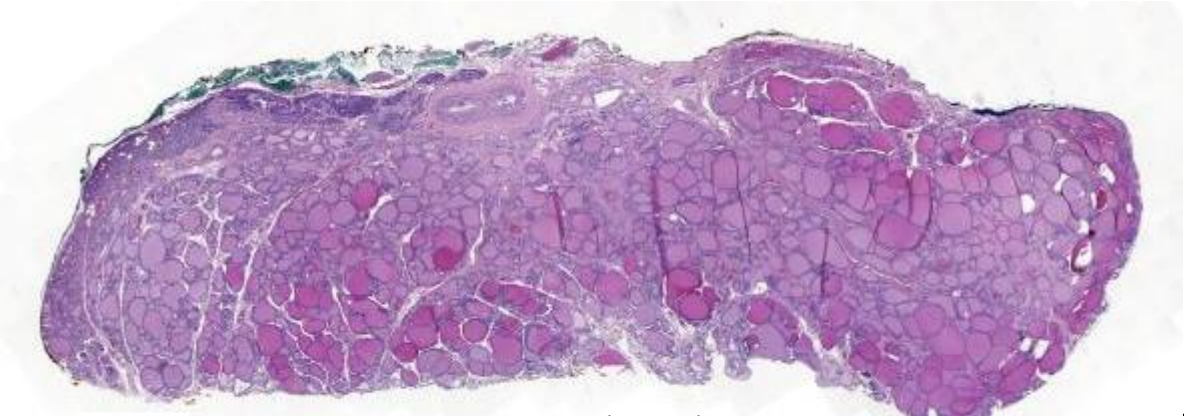

Control (5C-B)

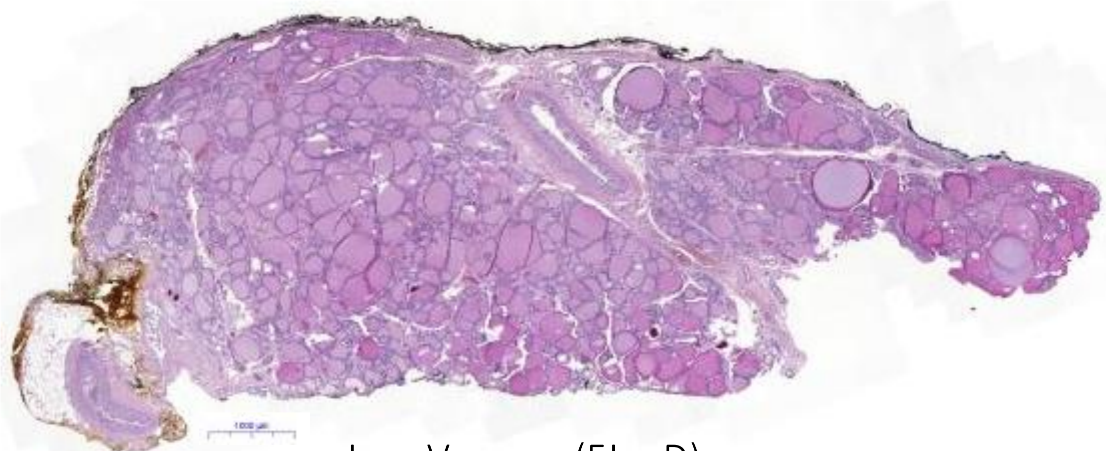

Low Vacuum (5Lo-D)

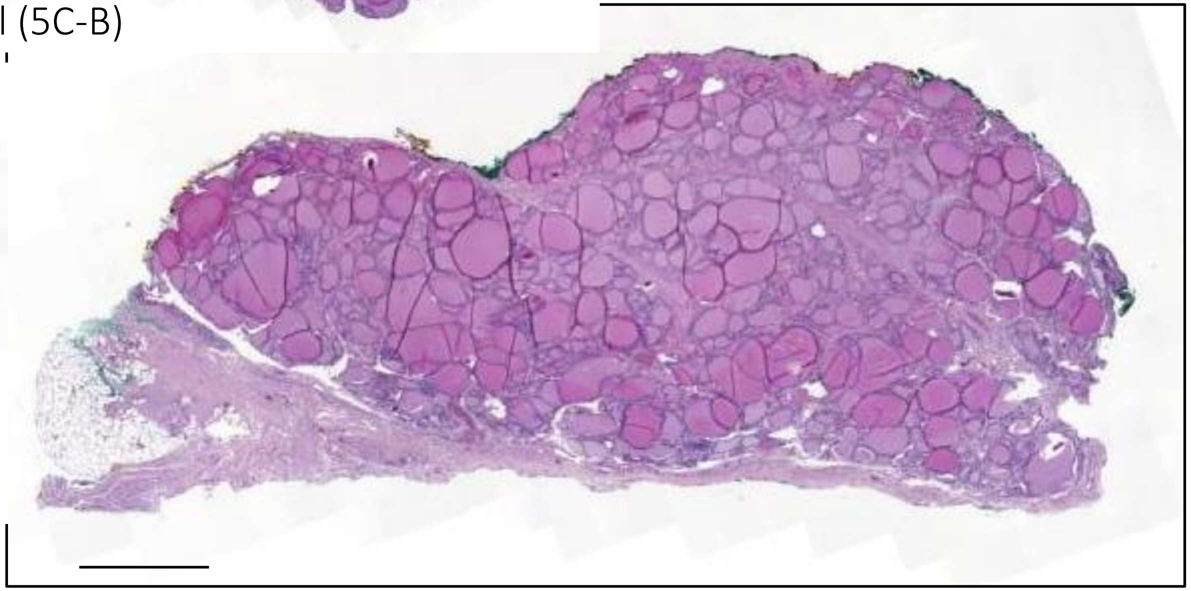

Full Vacuum

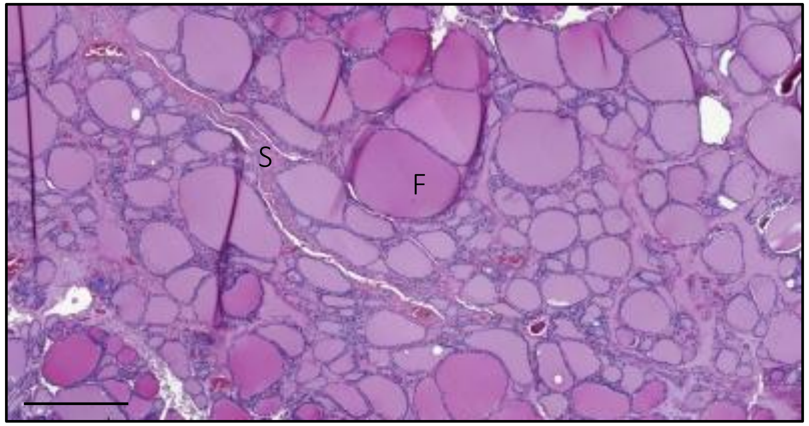

Control

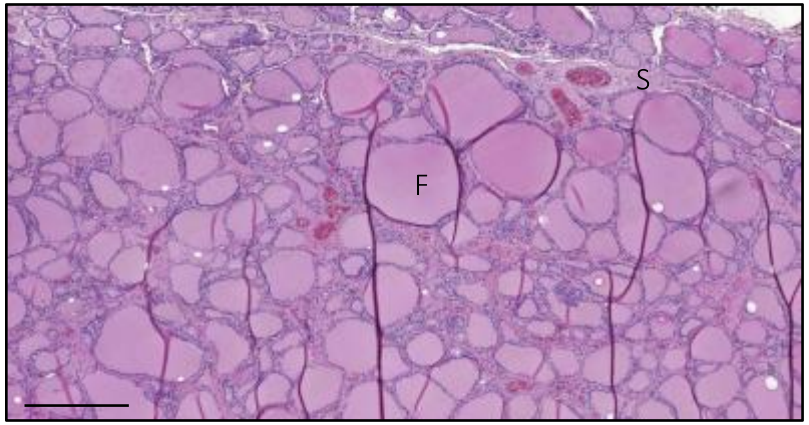

Low Vacuum

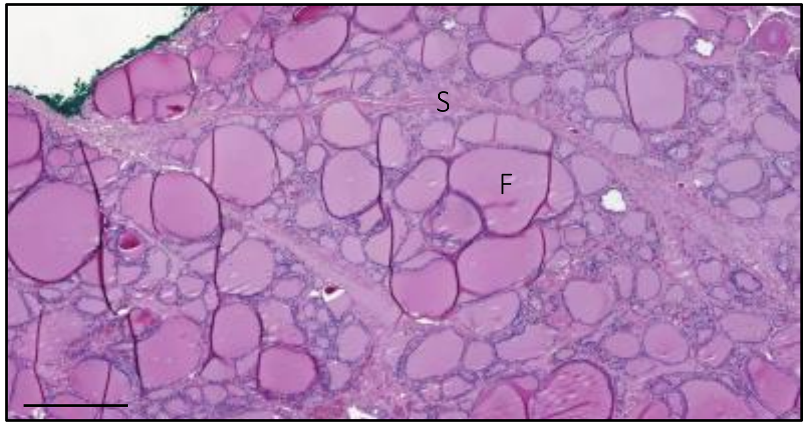

Full Vacuum

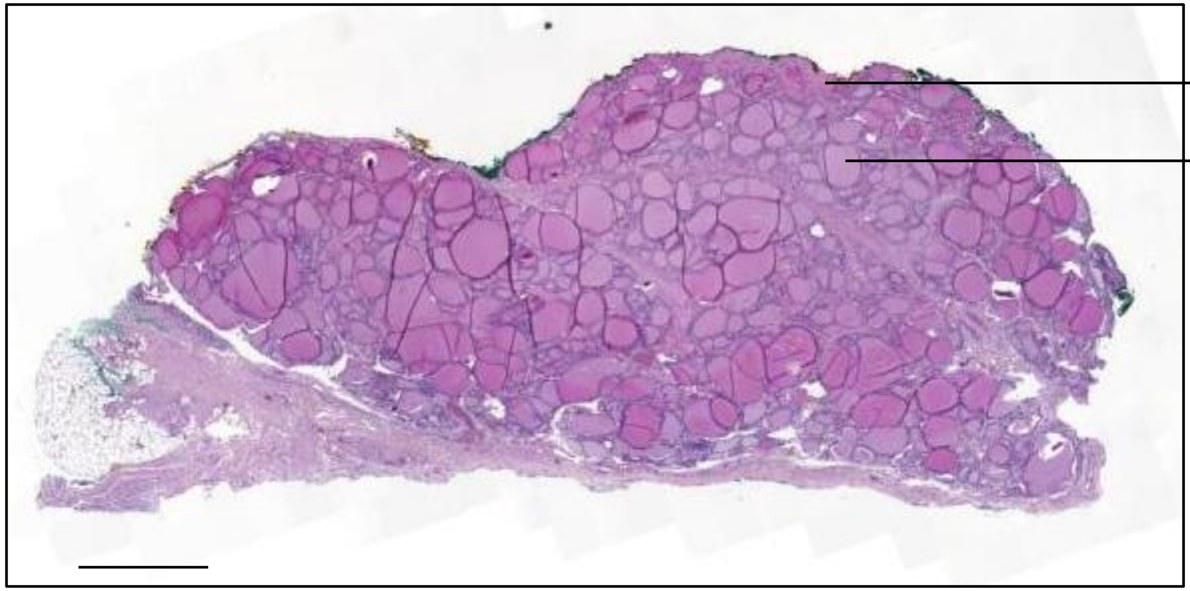

Full Vacuum

Histology slides – 6 (Adrenal)

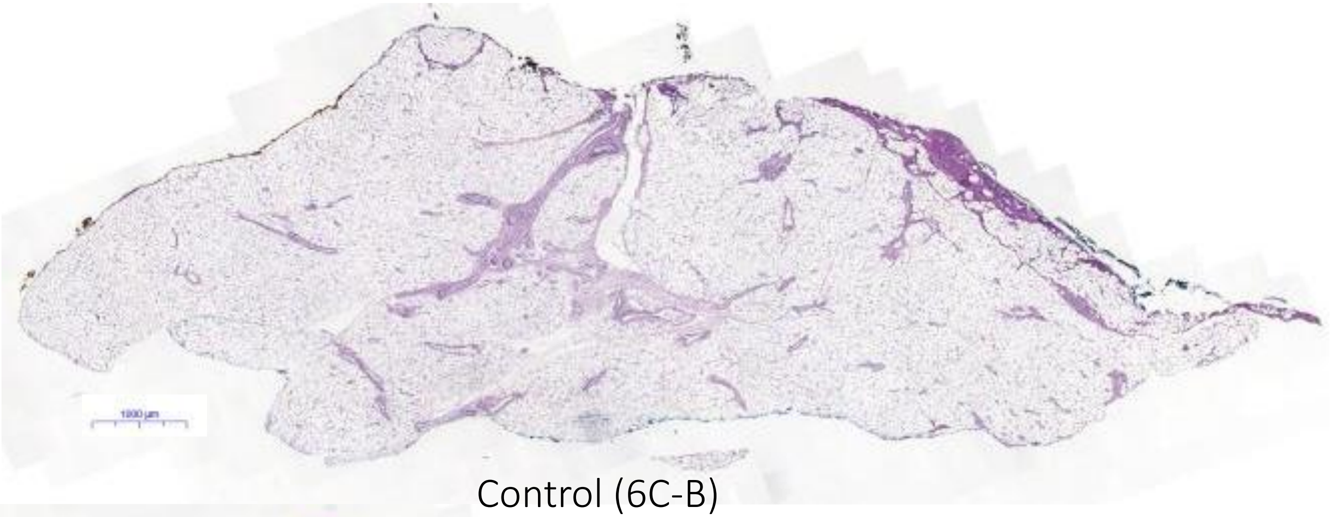

Control (6C-B)

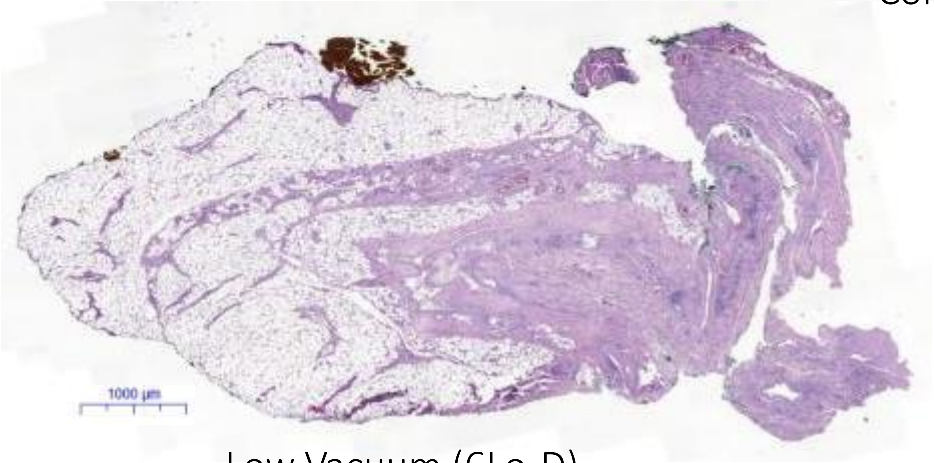

Low Vacuum (6Lo-D)

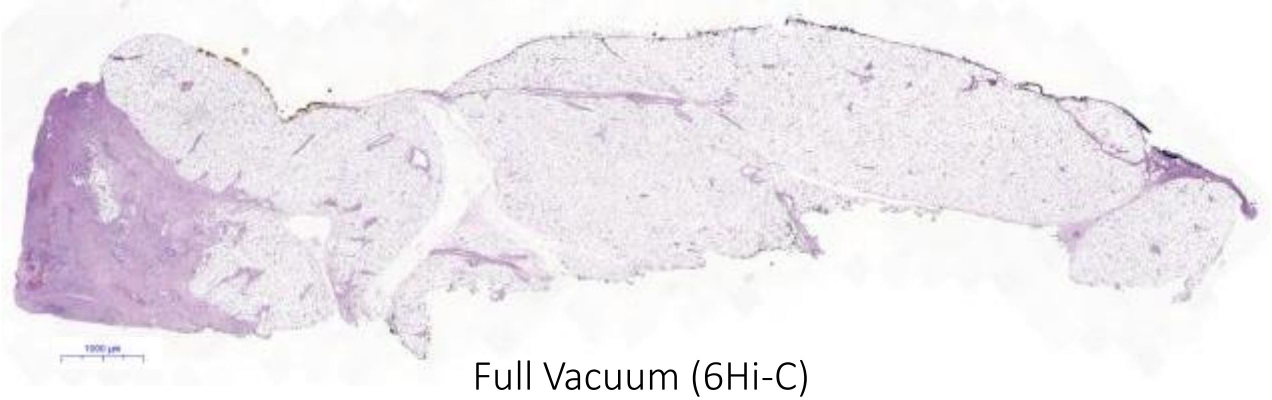

Full Vacuum (6Hi-C)

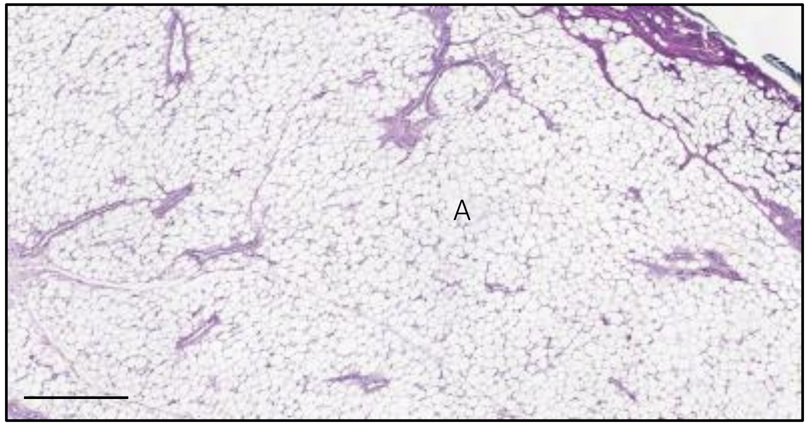

Control

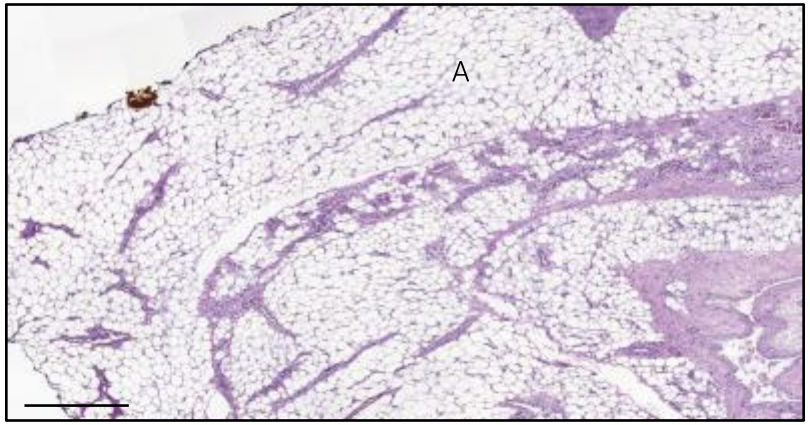

Low Vacuum

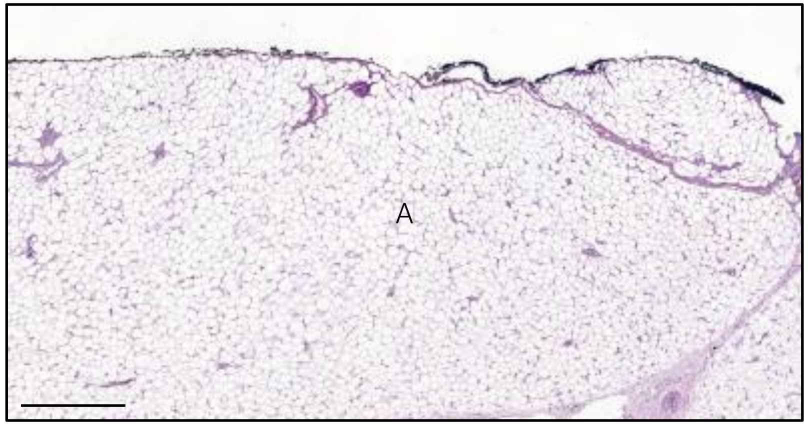

Full Vacuum

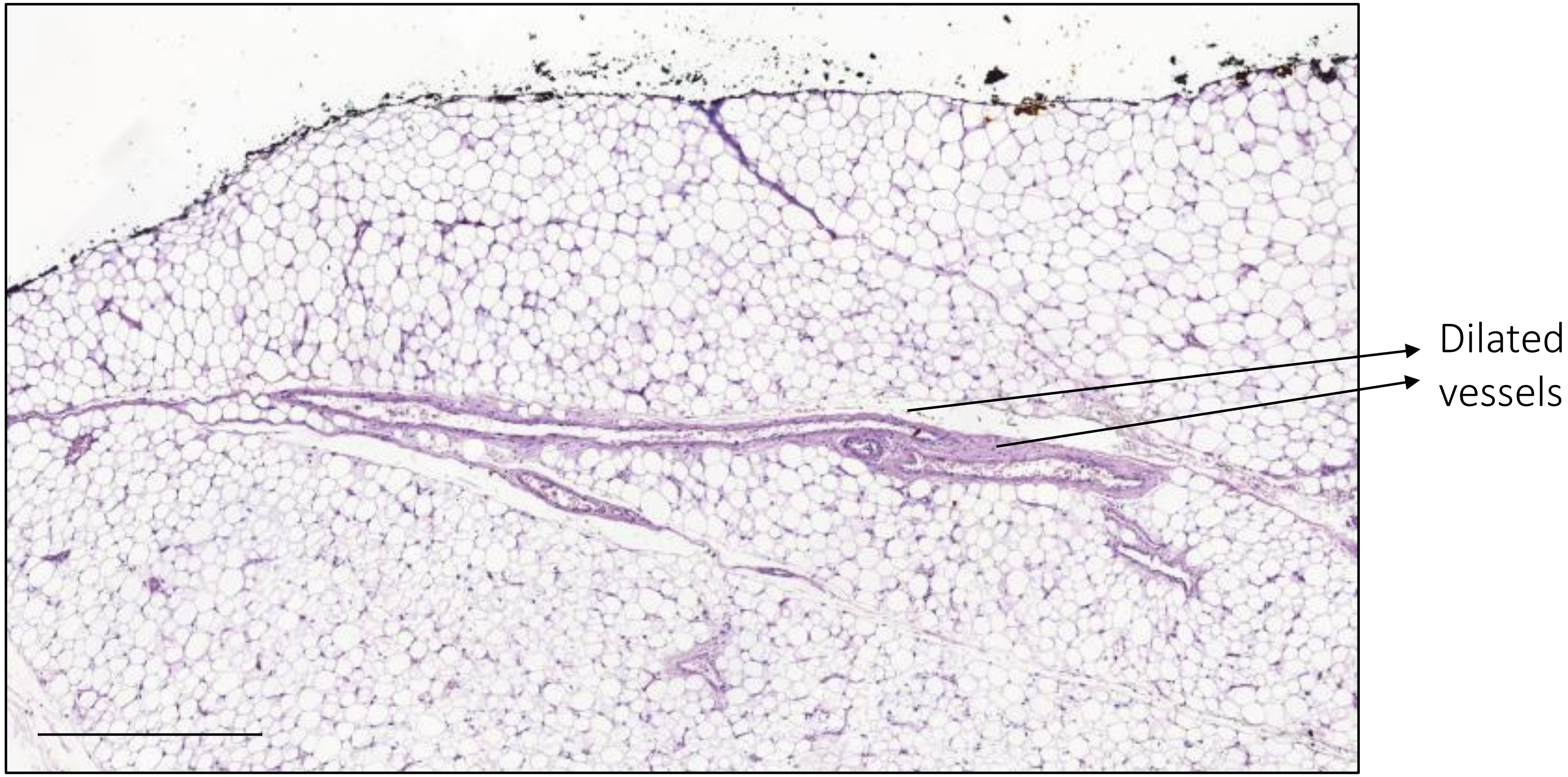
Dilated
vessels

Histology slides – 7 (Pancreas)

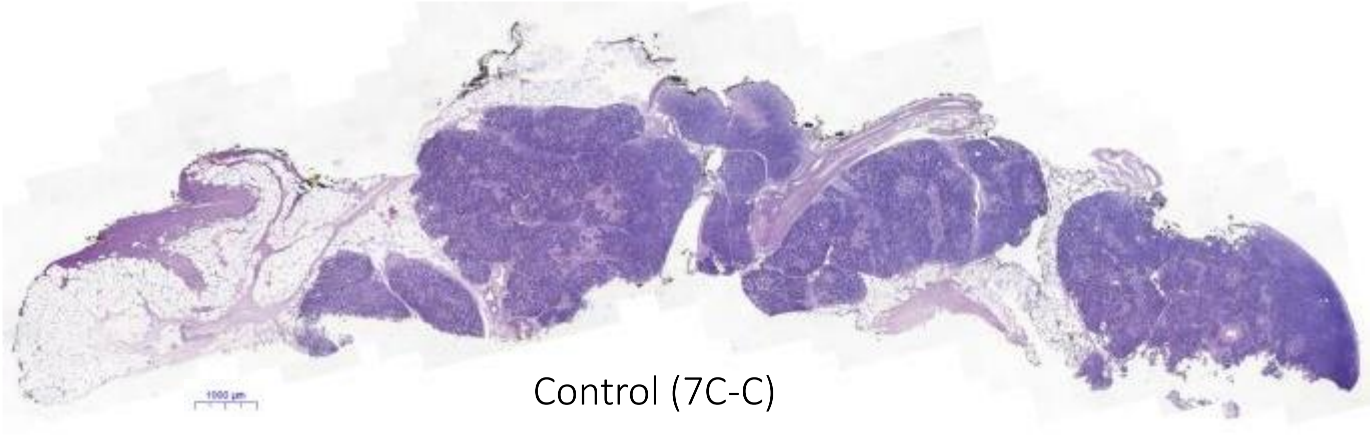

Control (7C-C)

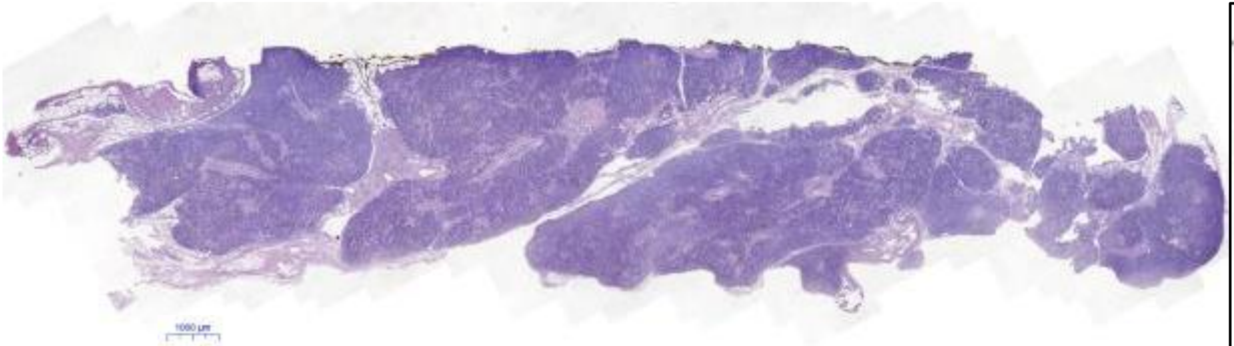

Low Vacuum (7Lo-C)

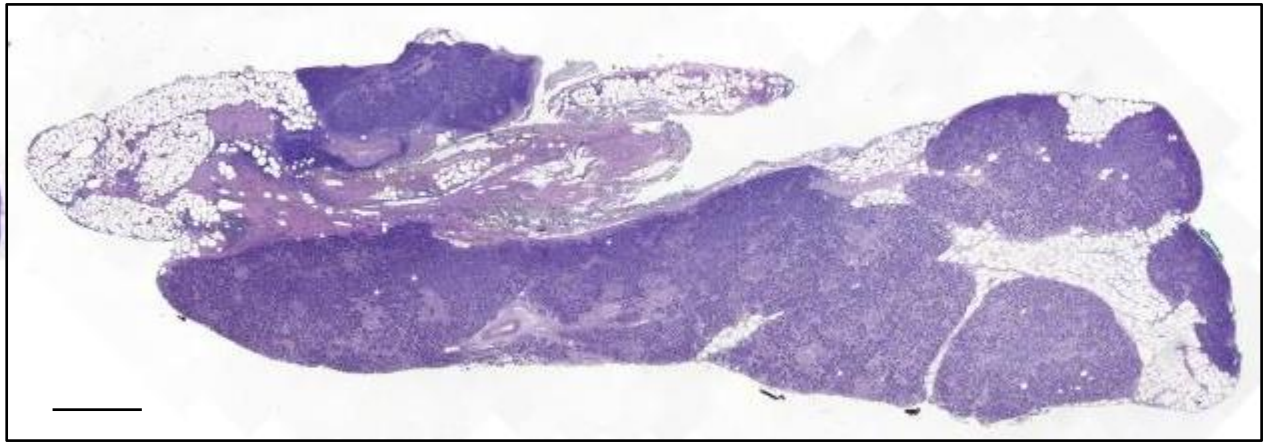

Full Vacuum

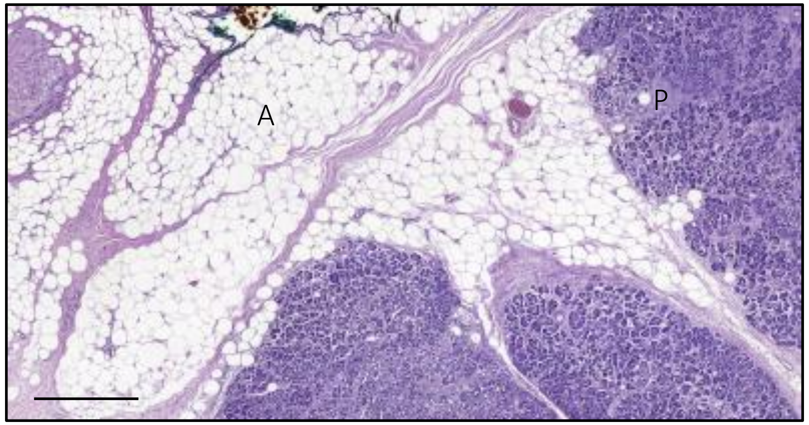

Control

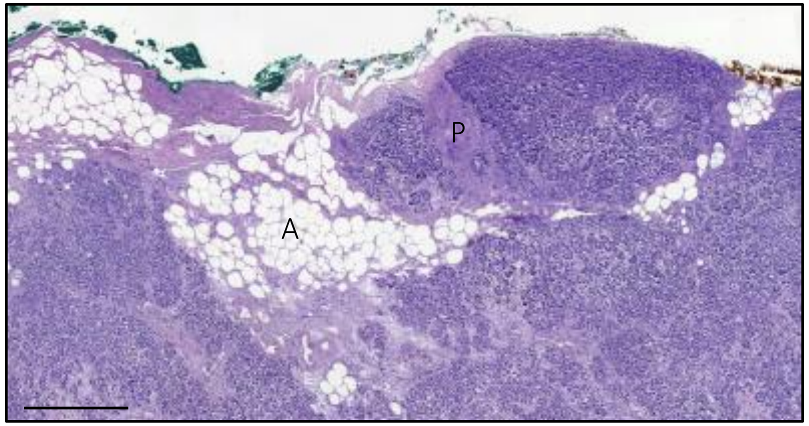

Low Vacuum

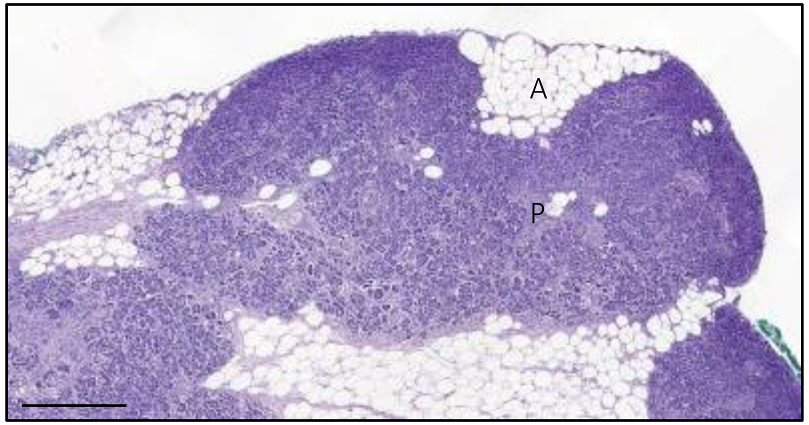

Full Vacuum

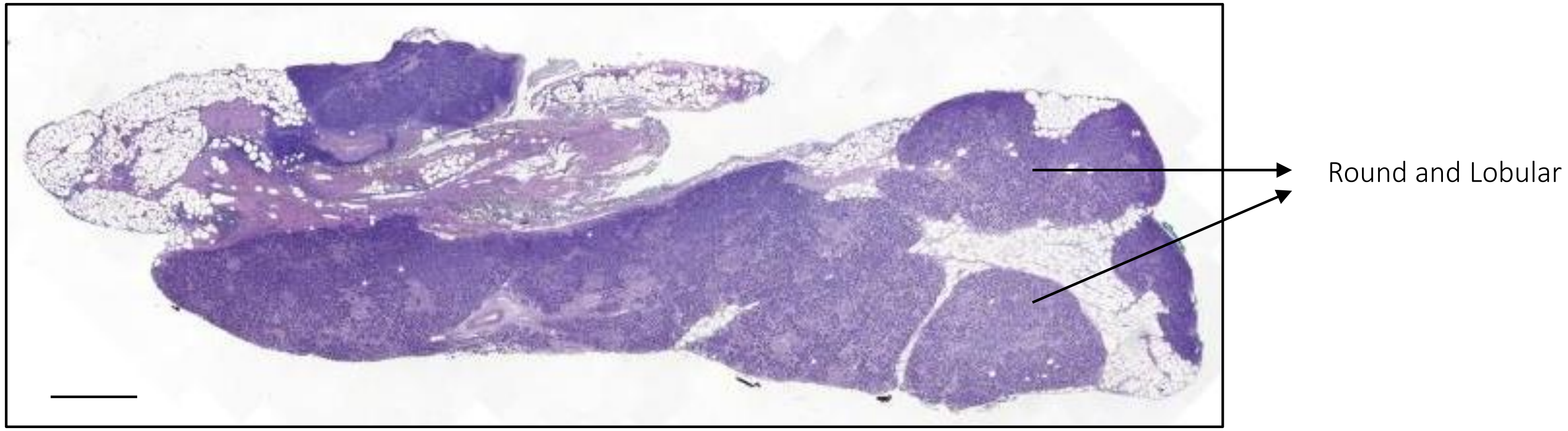

Full Vacuum

Histology slides – 8 (Liver)

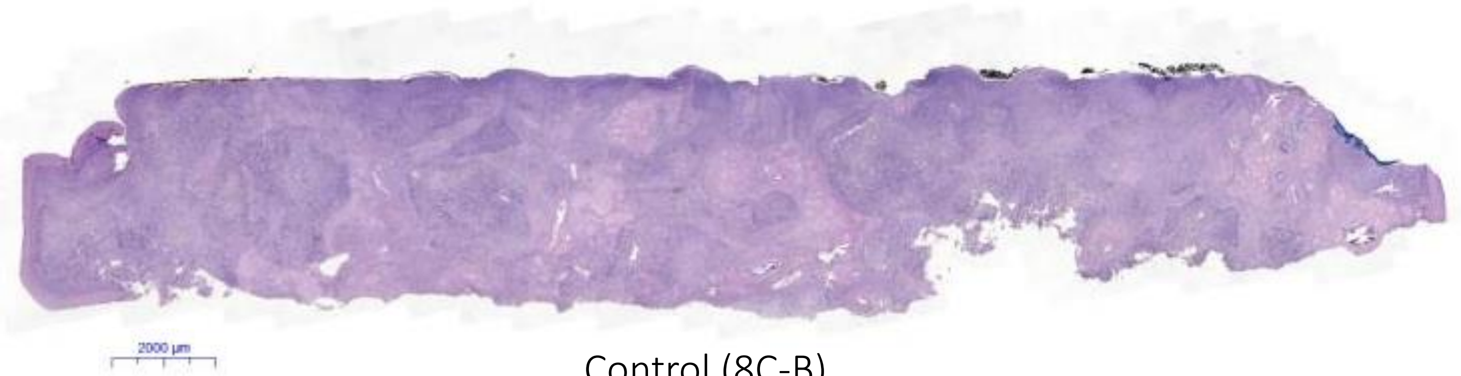

Control (8C-B)

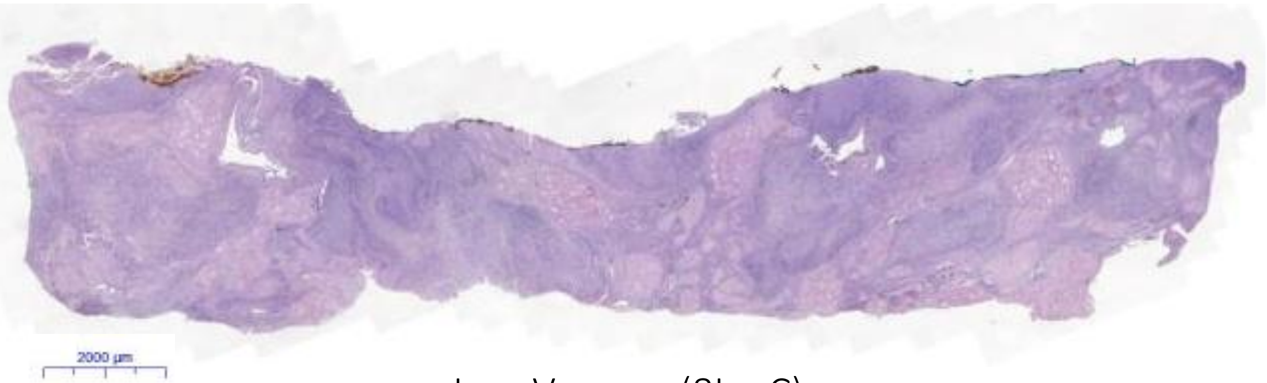

Low Vacuum (8Lo-C)

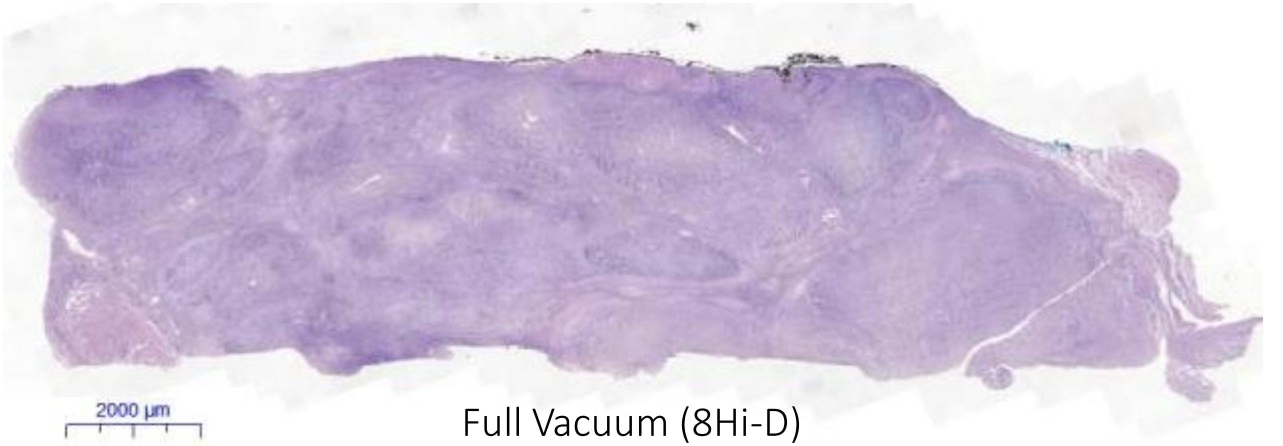

Full Vacuum (8Hi-D)

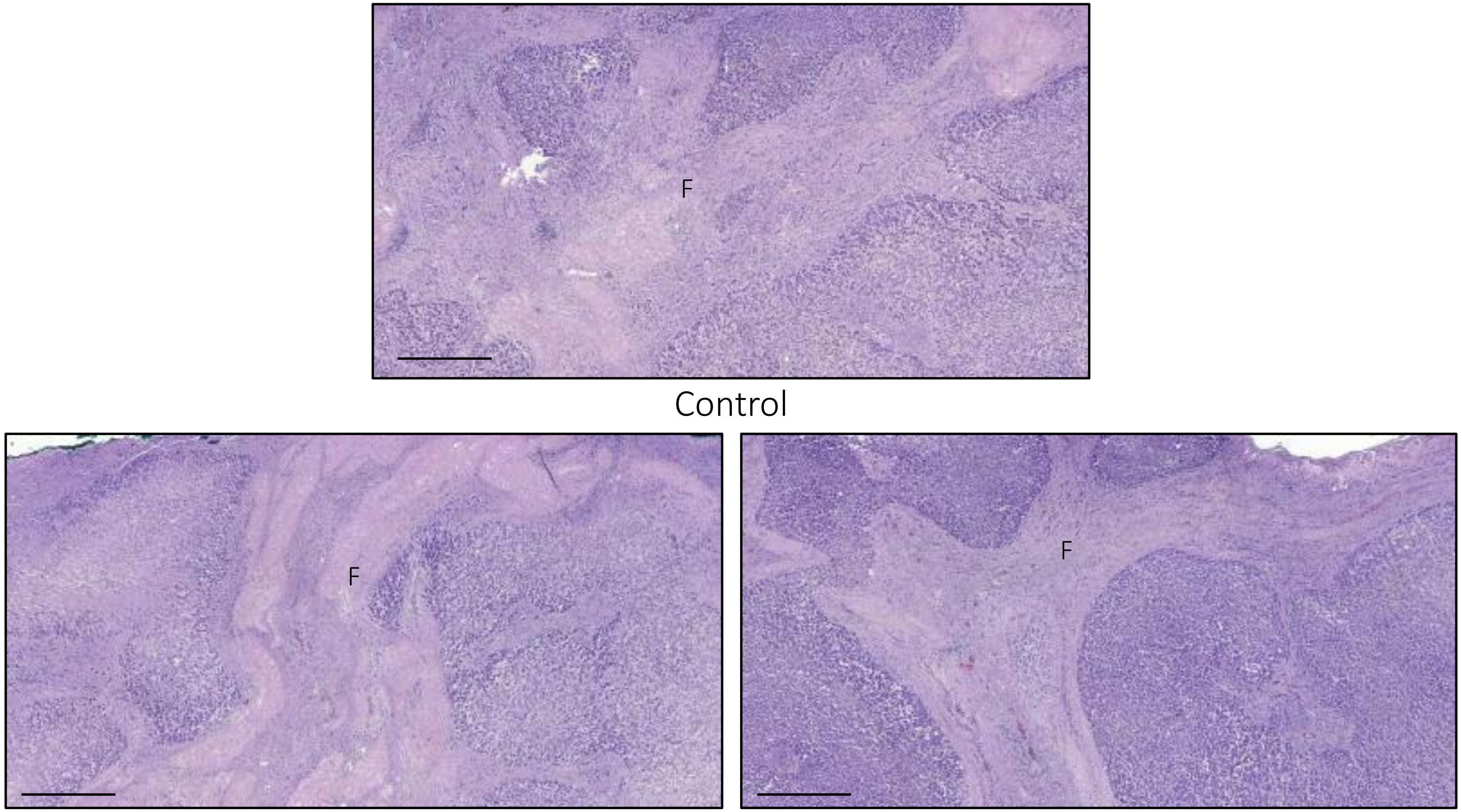

Control

Low Vacuum

Full Vacuum

Histology slides – 9 (Lung)

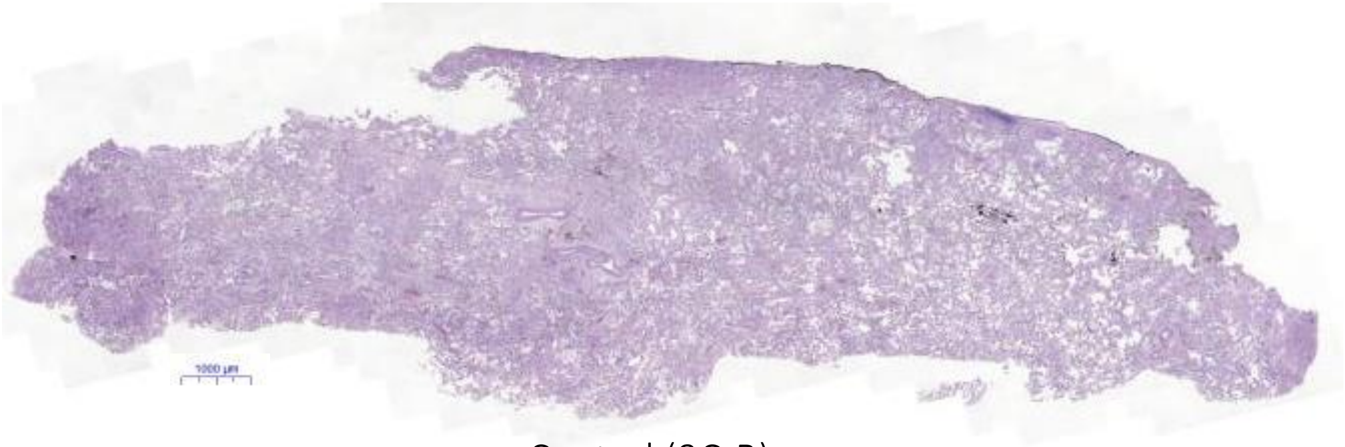

Control (9C-B)

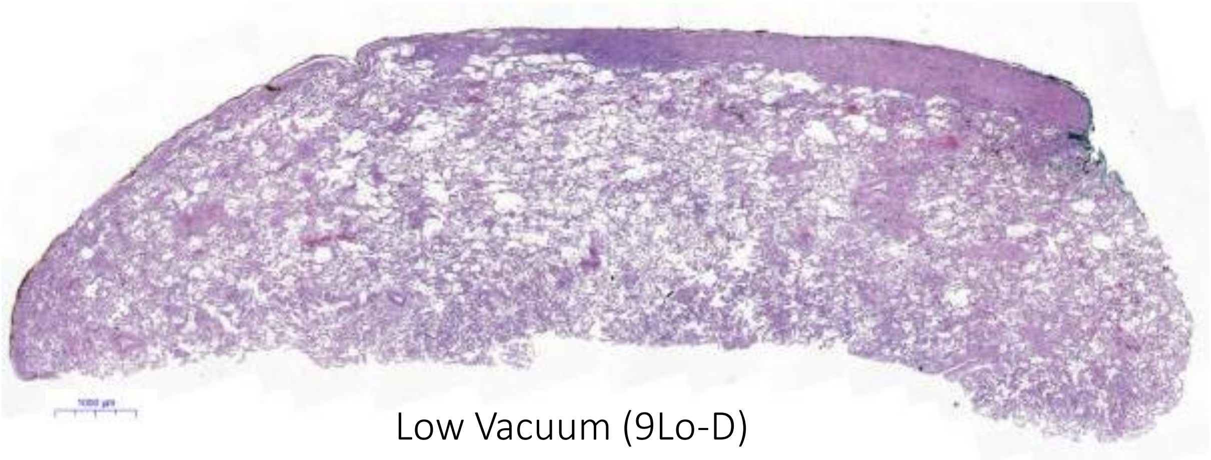

Low Vacuum (9Lo-D)

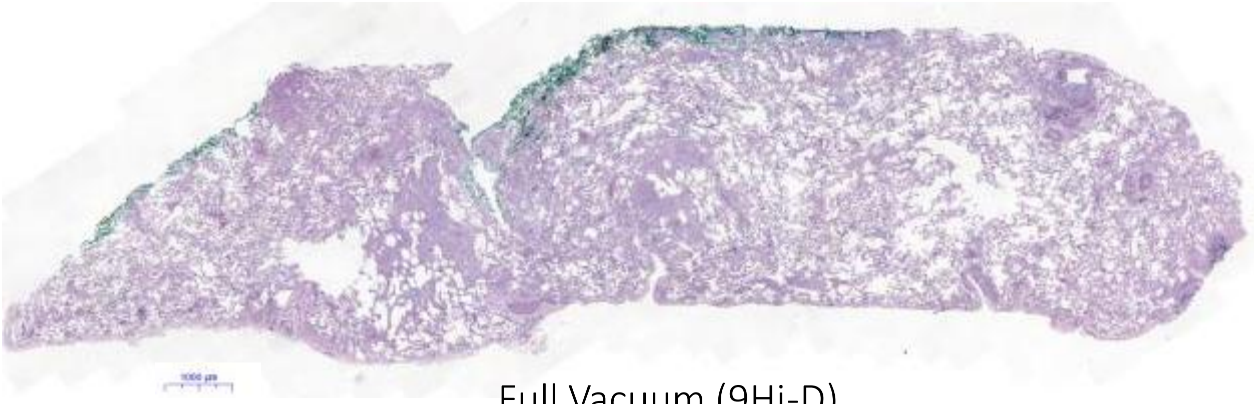

Full Vacuum (9Hi-D)

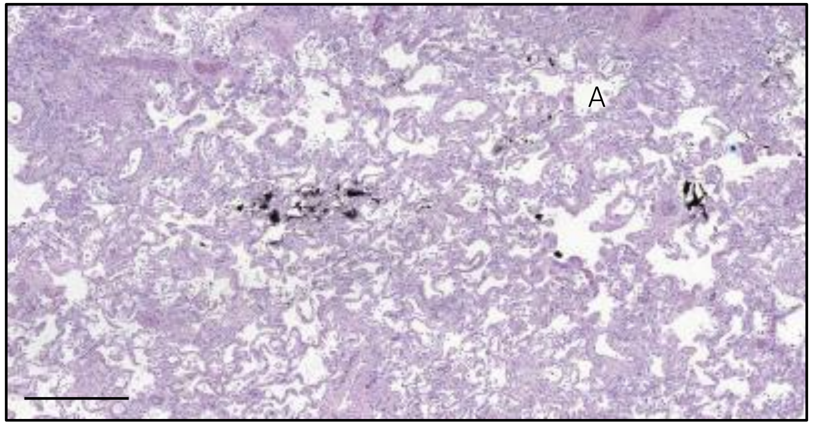

Control

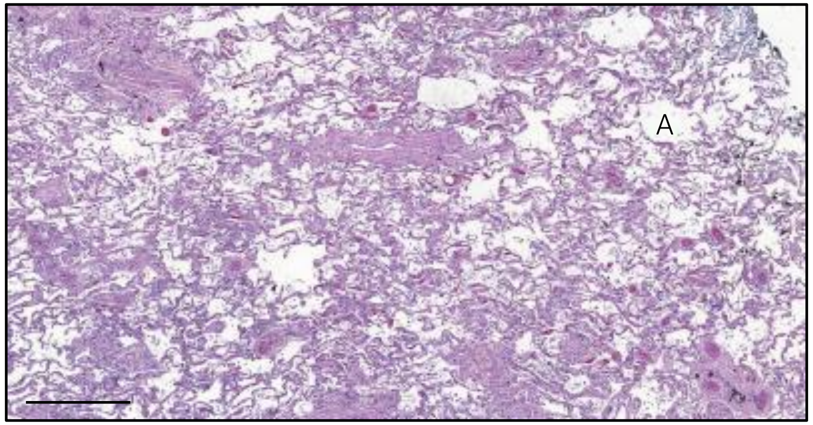

Low Vacuum

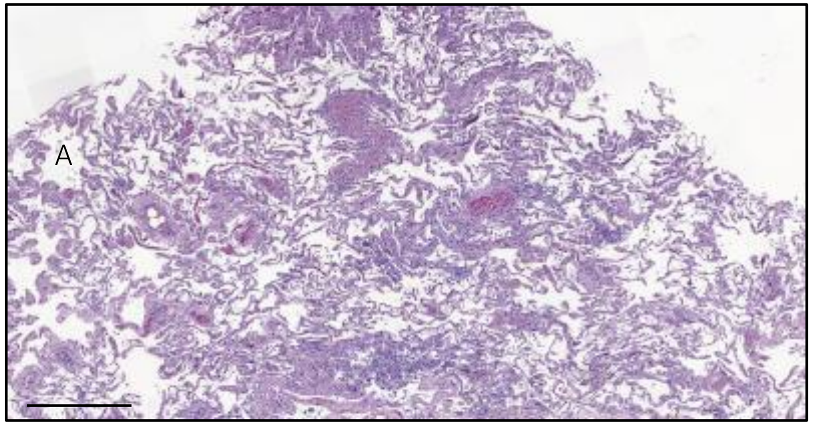

Full Vacuum

Histology slides – 10 (Colon)

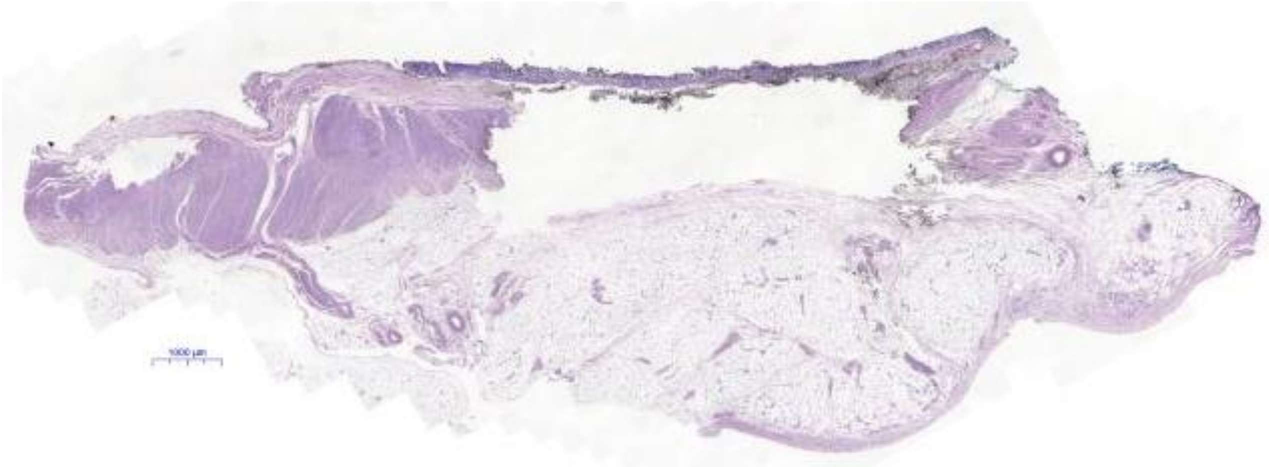

Control (10C-D)

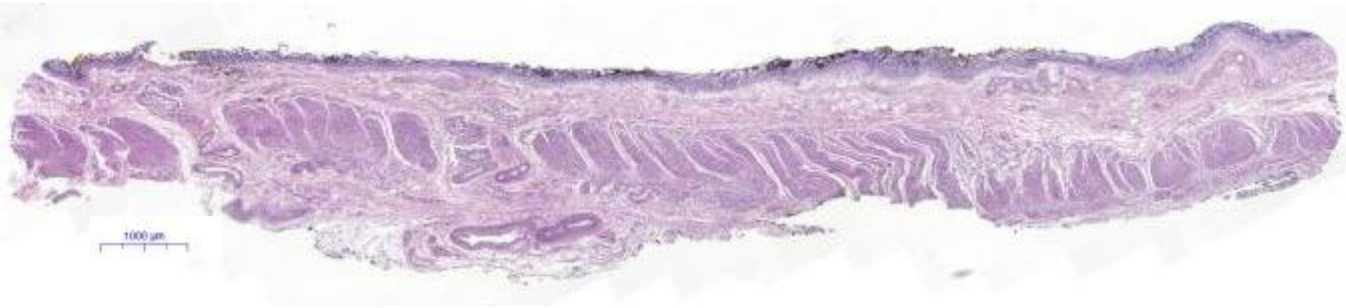

Low Vacuum (10Lo-D)

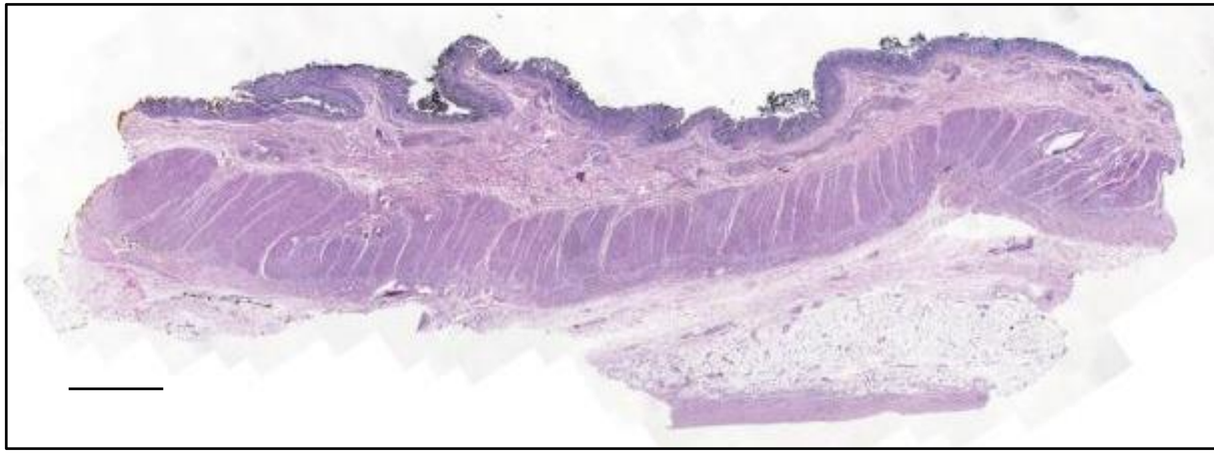

Full Vacuum

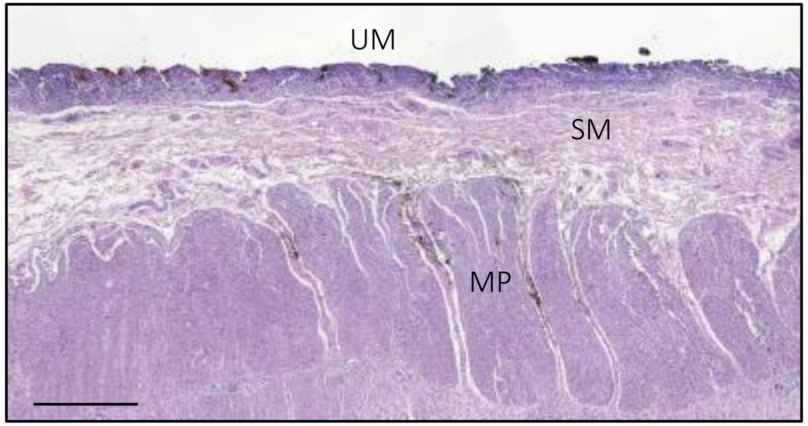

Control

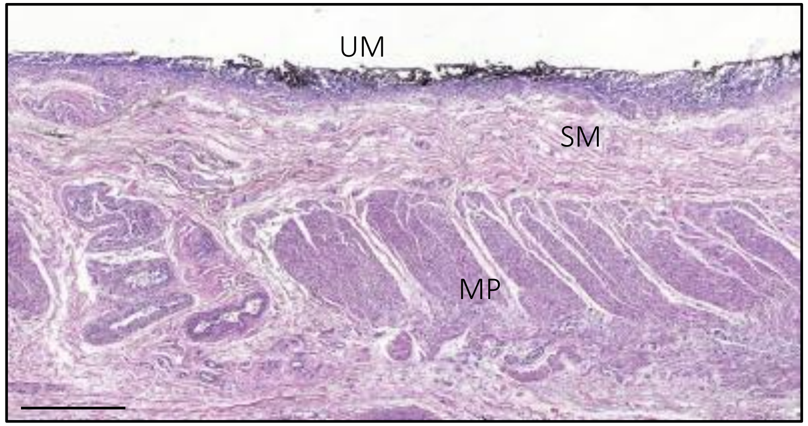

Low Vacuum

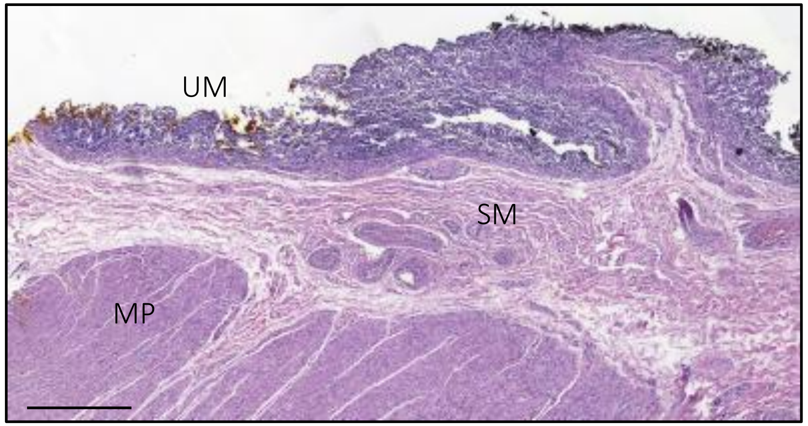

Full Vacuum

Papillae/villi not flattened or damaged

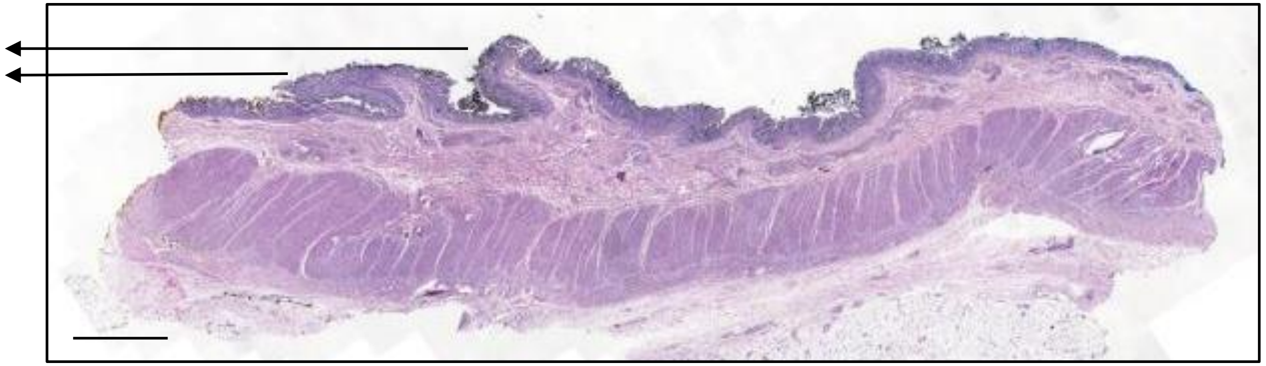

Full Vacuum
